# Lateralization in Hemiparkinsonian Rats Is Affected by either Deep Brain Stimulation or Glutamatergic Neurotransmission in the Inferior Colliculus

**DOI:** 10.1523/ENEURO.0076-22.2022

**Published:** 2022-07-27

**Authors:** Liana Melo-Thomas, Lars Tacken, Nicole Richter, Davina de Almeida, Catarina Rapôso, Silvana Regina de Melo, Uwe Thomas, Yara Bezerra de Paiva, Priscila Medeiros, Norberto Cysne Coimbra, Rainer Schwarting

**Affiliations:** 1 Behavioral Neuroscience, Experimental and Biological Psychology, Philipps-University of Marburg, D-35032 Marburg, Germany; 2 Center for Mind, Brain, and Behavior (CMBB), 35032 Marburg, Germany; 3 Behavioral Neurosciences Institute (INeC), Ribeirão Preto, 14050-220, São Paulo, Brazil; 4 Laboratory of Drug Development, Faculty of Pharmaceutical Sciences, University of Campinas, Campinas, São Paulo, 13083-865, Brazil; 5 Department of Morphological Sciences, State University of Maringá, Maringá 5790, 87020-900, Paraná, Brazil; 6 Thomas RECORDING, 35394 Giessen, Germany; 7 Laboratory of Neuroanatomy and Neuropsychobiology, Department of Pharmacology, Ribeirão Preto Medical School, University of São Paulo (FMRP-USP), Ribeirão Preto, SP 14049-900, Brazil; 8 NAP-USP-Neurobiology of Emotions Research Centre (NuPNE), Ribeirão Preto Medical School, University of São Paulo (FMRP-USP), Ribeirão Preto, SP 14049-900, Brazil; 9 Laboratory of Neurosciences of Pain & Emotions and Multi-User Centre of Neuroelectrophysiology, Department of Surgery and Anatomy, Ribeirão Preto Medical School, University of São Paulo, Ribeirão Preto, SP 14049-900, Brazil

**Keywords:** 6-OHDA lesion, deep brain stimulation, glutamate, inferior colliculus, medial forebrain bundle, Parkinson’s disease

## Abstract

After unilateral lesion of the medial forebrain bundle by 6-OHDA rats exhibit lateralized deficits in spontaneous behavior or apomorphine-induced rotations. We investigated whether such lateralization is attenuated by either deep brain stimulation (DBS) or glutamatergic neurotransmission in the inferior colliculus (IC) of Wistar rats. Intracollicular DBS did not affect spontaneous lateralization but attenuated apomorphine-induced rotations. Spontaneous lateralization disappeared after either the glutamatergic antagonist MK-801 or the agonist NMDA microinjections into the IC. Apomorphine-induced rotations were potentiated by MK-801 but were not affected by NMDA intracollicular microinjection. After injecting a bidirectional neural tract tracer into the IC, cell bodies and/or axonal fibers were found in the periaqueductal gray matter, superior colliculus, substantia nigra, cuneiform nucleus, and pedunculo-pontine tegmental nucleus, suggesting the involvement of these structures in the motor improvement after IC manipulation. Importantly, the side of the IC microinjection regarding the lesion (ipsilateral or contralateral) is particularly important, and this effect may not involve the neostriatum directly.

## Significance Statement

The inferior colliculus, usually viewed as an auditory structure, when properly manipulated may counteract motor deficits in parkinsonian rats. Indeed, the present study showed that either 30 Hz deep brain stimulation or glutamatergic neural network in the inferior colliculus reduced body asymmetry induced by a medial forebrain bundle unilateral 6-OHDA lesion in rats, an animal model of parkinsonism. Understanding how glutamatergic mechanisms in the inferior colliculus influence motor control, which is classically attributed to the basal nuclei circuitry, could be useful in the development of new therapeutics to treat Parkinson’s disease and other motor disorders.

## Introduction

Parkinson’s disease (PD) is the second most common neurodegenerative disorder, and is characterized by resting tremor, bradykinesia, akinesia, and postural instability. It was only in 1960 after Ehringer and Hornykiewicz (1960) published their landmark article that PD was related to a profound loss of the pigmented dopamine-producing neurons in the substantia nigra pars compacta (SNpc) leading to a reduction of dopamine (DA) in the neostriatum. Although pharmacological DA replacement therapy or deep brain stimulation (DBS) typically targeting motor regions of the basal nuclei (Perlmutter and Mink, 2006) can alleviate some PD symptoms, patients still report side effects (Marsden and Parkes, 1977; Okun et al., 2005; Zheng et al., 2010; Simsek and Anderson, 2015; Højlund et al., 2017). In this respect, searching for a new therapeutic and nonconventional DBS target to relieve motor impairment in patients with PD may be of interest. In this sense, the inferior colliculus (IC) raises as a putative candidate. In addition to being a midbrain tectum structure known for its pivotal role in acoustic processing, the IC provides a node in a highly interconnected sensory, motor, and cognitive network dedicated to synthesizing a higher-order auditory percept (Gruters and Groh, 2012). Indeed, it is distinguished from other auditory nuclei in the brainstem by its output connections to motor pathways and movement coordination systems (Casseday and Covey, 1996; Caggiano, et al., 2018). There is also evidence in rats that motor systems project to the IC, such as projections from the substantia nigra pars lateralis (SNpl; Olazábal and Moore, 1989) and from the globus pallidus (Moriizumi and Hattori, 1991). In this line of evidence, it was demonstrated that either DBS or microinjection of glutamatergic antagonists (AP7 or MK-801) into the IC attenuated haloperidol-induced catalepsy in awake rats (Melo et al., 2010; Medeiros et al., 2014; Melo-Thomas and Thomas, 2015; Engelhardt et al., 2018; Ihme et al., 2020). Catalepsy in rodents is a state of immobility attributed mainly to the blockade of D_2_ DAergic receptors in the striatum induced by systemic or intrastriatal administration of haloperidol (Hornykiewicz, 1973; Sanberg, 1980; Wadenberg et al., 2001). Such catalepsy is used as an experimental model of akinesia, a lack of spontaneous motor activity that is common in PD.

Although haloperidol-induced catalepsy has contributed to the discovery of symptomatic drugs for PD, it produces only an acute motor impairment. In a condition like PD, patients experience chronic motor impairments; therefore, it is important to investigate the effectiveness of treatment on long-term symptoms (i.e., lesions), as it has already been demonstrated in mice with MPTP (1-methyl-4-phenyl-1,2,3,6-tetrahydropyridine) lesions (Melo-Thomas et al., 2018) and receiving intracollicular DBS. Here, this research was extended to rats, and it was asked whether either DBS or glutamatergic neural substrate in the IC affects chronic motor impairments induced by 6-hydroxydopamine (6-OHDA). Injection of this catecholaminergic neurotoxin in the medial forebrain bundle (MFB), for instance, results in denervation of the dopaminergic nigrostriatal pathway and is considered as a classic chronic animal model for parkinsonism (Ungerstedt, 1968; Ungerstedt and Arbuthnott, 1970; Salari and Bagheri, 2019), since it leads to a motor deficit that is stable over time. After receiving unilateral microinjection of 6-OHDA in the MFB, rats exhibit lateralized deficits in spontaneous behavior, such as ipsiversive (toward the side of lesion) rotations (Ungerstedt and Arbuthnott, 1970; Schwarting and Huston, 1996a). Systemic challenge with the DA receptor agonist apomorphine (APO) induces contraversive (away from side of lesion) rotation, as a result of DA receptor supersensitivity in the DA-depleted hemisphere (Schwarting and Huston, 1996b; Deumens et al., 2002). This is particularly important since contraversive rotations in 6-OHDA-lesioned rats are closely related to dyskinesia, one of the most marked disabilities observed during the late phase of PD (Konitsiotis and Tsironis, 2006; Lane et al., 2006). Another important point is that this rotatory behavior is highly correlated with the amount of thigmotactic scanning (locomotion along the wall with the vibrissae in contact with it; Schwarting et al., 1991). For instance, a rat with a severe 6-OHDA lesion of the right nigrostriatal pathway spontaneously locomotes clockwise (ipsiversive rotations) along the walls of an open-field, but has more scanning with the left side of the body (the dominant side). Contrarily, after receiving APO, this same rat rotates anticlockwise not only because it exhibits a contraversive body asymmetry, but also because it shows more scanning (more exploration) with the side of the body ipsilateral to the lesion (now the dominant side; Thal et al., 1979; Schwarting et al., 1991). The present study was undertaken to investigate whether (1) intracollicular DBS can ameliorate neurochemical lesion-induced behavioral asymmetries as assessed by spontaneous or APO-induced contraversive behavior; (2) whether such lateralization in hemiparkinsonian rats is attenuated by glutamatergic manipulations in the IC; (3) whether glutamatergic manipulations in the IC affect neural activity in the neostriatum in anesthetized rats; and (4) which motor structures receive projections from the IC.

## Materials and Methods

### Animals

One hundred twenty-six male Wistar rats, weighing 290–300 g on the day of surgery, were used in four distinct experiments. Ninety-seven rats were used for behavioral experiments, and 23 rats were used for electrophysiology (*n* = 19) and the neural tract tracer (*n* = 4) study. A total of six rats was excluded from our study because of electrode misplacement or because they did not present contraversive rotations after apomorphine administration. Rats were housed in Plexiglas-walled cages in groups of five by cage and had *ad libitum* access to water and food (room temperature, 22 ± 1°C; humidity, 55 ± 5%; 12 h light/dark cycle). After surgery, rats were kept individually for 1 day and were later housed in pairs in Macrolon type III cages with extra high acrylic covers (length, 22 cm; width, 38 cm). All protocols were performed in accordance with the European Manual and approved by the local ethics committee (TVA G53-2016, Tierschutzbehörde, Regierungspräsidium; behavioral experiments) or with the recommendations of the Brazilian Society for Neuroscience and Behavior, which are based on the of the US National Institutes of Health *Guidelines for the Care and Use of Laboratory Animals* (publication No. 85–23, revised 1985), and were approved by the Ethics Committee of the University of São Paulo (CEP: 0091/12; electrophysiology and neural tract tracer experiments).

### Drugs and doses

6-OHDA (Sigma-Aldrich) 12 μg/μl was dissolved in 0.02% ascorbic acid/physiological saline solution. The following drugs were dissolved in physiological saline: NMDA (Sigma-Aldrich) at 30 nmol/0.5 μl; (+)−5-methyl-10, 11-dihydro-5H-dibenzo (a,d)-cyclohepten-5,10-imine (MK-801; Research Biochemicals International) at 30 nmol/0.5 μl; and APO (Teclapharm) at 0.5 mg/kg were used. The drugs and doses used here were based on those used in previous studies (Ciucci et al., 2007; Melo et al., 2010; Medeiros et al., 2014; Tonelli et al., 2018).

### Stereotaxic surgery

Rats were anesthetized with 2% isoflurane (Baxter Deutschland) and fixed in a stereotactic frame (TSE Systems). Ophthalmic ointment Bepanthen (Bayer Vital) was applied to prevent eye drying, and 0.5 ml of xylocaine (Dentsply De Trey) was injected subcutaneously under the shaved scalp to minimize discomfort.

#### 6-OHDA and sham lesions

Hemiparkinsonian rats (lesion group) were created by lesioning the right MFB as described previously (da Cunha et al., 2008). Briefly, a burr hole was drilled above the MFB, allowing 1 μl of 6-OHDA (12 μg/μl) to be injected into the right MFB according to the following coordinates (relative to bregma): anteroposterior (AP), 1.9 mm; mediolateral (ML), −1.9 mm; dorsoventral (DL), 7.2 mm (from the dura mater; Paxinos and Watson, 2007). 6-OHDA was freshly prepared before each operation and kept in the dark throughout each surgery. Injections were made using a stainless steel dental needle (30 gauge; Miraject) connected by a polyethylene tube to a 10 ml Hamilton syringe, at a rate of 1 μl/min controlled by a microinjection pump (model sp101i, WPI). After injection, the injector needle was left in place for 3 min to allow local toxin diffusion. The sham group received the vehicle solution (1 μl of 0.02% ascorbic acid in physiological saline) and was used as a control. Immediately after toxin or physiological saline microinjection in MFB, the same rats were also implanted with an electrode or microinjection guide cannula in the IC.

#### Electrode implantation

Rats (*n* = 53) were implanted unilaterally into the IC with a microelectrode unit, consisting of a stimulation electrode (90% platinum wire; 10% iridium wire; core diameter, 125 μm; outer diameter, 150 μm; impedance, <10 kΩ; Thomas RECORDING), connected to a contact plate and a platinum wire reference electrode (shaft diameter, 100 μm). The electrode was inserted into the right or left side using the following coordinates aiming at the central nucleus (CN) of the IC (Paxinos and Watson, 2007) with distance from bregma as reference: anteroposterior, +8.6 mm; mediolateral, ±1.5 mm; dorsoventral, +4.5 mm. Although the same coordinates were used for all rats, after histologic analysis rats with electrode tips at the most dorsal part of the CN of the IC (dCN) and the most ventral part of the CN of the IC (vCN) were separated into two different subgroups. Each rat had an electrode implanted in the IC on the same side or on the opposite side of the lesion. The electrode was fixed to the skull with four stainless steel screws covered with ultraviolet adhesive (Duo-Link Universal, BISICO) and acrylic resin. A protective cap was used to cover the electrode contacts.

#### Cannula implantation

Rats (*n* = 44) were implanted bilaterally with stainless steel guide cannulae (22 gauge; length, 13 mm; Thomas RECORDING) aimed at the following IC coordinates (Paxinos and Watson, 2007) with λ as reference: anteroposterior, +1.0 mm; mediolateral, ±1.5 mm; dorsoventral, +4.5 mm. The cannulae were fixed to the skull and covered as described in the case of electrode fixation. A stiletto put inside the guide cannulae prevented obstruction. After surgery, rats were kept individually for 1 day and were later housed in pairs in Macrolon type III cages with extra-high acrylic covers (length, 22 cm; width, 38 cm). Postoperative care included buprenorphine (0.05 mg/kg; Titolare A.I.C.) injected subcutaneously every 12 h for 72 h, and body weights and general health status were monitored daily up to the end of the behavioral experiments. Laboratory conditions were standardized (temperature, 23°C; humidity, 40–60%; 12 h light/dark cycle), and free access to water and food was provided. The behavioral tests started 7 day after surgery.

### Behavioral assessments

#### Elevated plus maze test

Although behavioral asymmetries are usually investigated in open-field tests arenas, previous evidence showed that they can also be investigated using an elevated plus maze (EPM; Schwarting and Borta, 2005), which is classically used to assess anxiety-related behaviors. Behavioral asymmetries in an EPM can be assessed, since the animal often has decision points to turn into an arm to its left or right, and, based on their turning asymmetries, one could expect that rats with unilateral 6-OHDA lesions turn more toward the ipsilateral side (since it neglects the contralateral side). For that, to encourage exploration, anxiety was reduced by keeping the light conditions darker than usual (a dim red light ∼10 lux in the testing room), based on the study by Morato and Castrechini (1989), who showed that rats tested under low light intensity increased the number of entries and the time spent in open arms. Using this approach, our goal was to investigate whether asymmetry in hemiparkinsonian rats is affected by intracollicular 30 Hz DBS.

The apparatus consisted of two open arms (50 × 10 cm) and two closed arms (50 × 10 cm, with 40 cm high walls) extending from a central platform elevated 50 cm above the floor. Behavior was monitored by a video camera (model WVBP330/GE, Panasonic) from ∼150 cm above the EPM. Before each test, the EPM apparatus was cleaned using a 0.1% acetic acid solution followed by drying. Electrical currents were delivered (30 Hz; current amplitude, 600 μA; pulse width, 100 μs) by connecting the implanted stimulation electrodes to a pulse generator using a tethered system (model STG3008-FA, Multichannel Systems) through a slip ring commutator (model SL12C, Bilaney Consultants). The stimulation procedure and parameters used here were based on previous studies from our group (Engelhardt et al., 2018; Ihme et al., 2020). The rats were randomly assigned to the following groups: Lesion DBS (*n* = 15), Lesion No DBS (*n* = 18), Sham lesion DBS (*n* = 12), and Sham lesion No DBS (*n* = 8). The stimulation cable was connected to the implanted electrode, and rats were first stimulated for 5 min in a clean home cage placed near the EPM. Thereafter, the rats were placed on the EPM, with stimulation being continued throughout testing. Specifically, each rat was placed onto the central platform of the EPM with its head facing one of the open arms and then allowed to freely explore the open and closed arms for 5 min according to a protocol previously reported (Pellow et al., 1985; Melo-Thomas et al., 2017). The procedure in No-DBS controls was identical except that no current was administered. The following behaviors were assessed: (1) the number of entries into and time spent on the open and closed arms; (2) the number of turns (90°; when approaching the center, the side chosen to change from open to closed arms, or vice versa) contraversive and ipsiversive to the lesion side; (3) the number of half rotations (180°) and rotations (360°) contraversive and ipsiversive to the lesion side (at both open and closed arms or at the center); and (4) the number of risk assessments contraversive and ipsiversive to the lesion side. Here, risk assessment was defined as aborted attempts to enter into open arms, which includes scanning, stretch/attend postures (when the rat stretches to its full length with its forepaws, keeps its hindpaws in the same place, and then turns back) and flat back approach (locomotion during which the animal stretches to its full length and cautiously moves forward; Griebel et al., 1997; Coimbra et al., 2017; Ferreira-Sgobbi et al., 2022). Each rat was tested only once on the EPM.

#### Apomorphine-induced rotation test

The goal was to investigate the effect of intracollicular glutamatergic manipulations or 30 Hz DBS to counteract APO-induced rotation behavior in hemiparkinsonian rats. Rotational asymmetry was assessed using an activity box (40 × 40 × 40 cm) under red light (∼30 lux).

##### Effect of 30 Hz intracollicular DBS

The same stimulation parameters as described for the EPM test were used. For assessing rotatory behavior under intracollicular 30 Hz DBS, immediately after each rat had received APO (0.5 mg/kg, s.c.), the stimulation cable was connected to the implanted electrode and rats were placed into the arena under continuous stimulation during 30 min. Behavior was monitored over 30 min by a video camera (model WVBP330/GE, Panasonic) positioned centrally 1 m above the arena. Here, 25 rats (Lesion, *n* = 18; Sham lesion, *n* = 7) that had participated in the EPM test were reused. During data analysis, lesion rats were assigned (dCN, *n* = 6; vCN, *n* = 12); then, vCN rats were reassigned to positions contralateral and ipsilateral to lesion (*n* = 6 each). Sham lesion rats were not compared considering electrode placement in the IC as a factor, because the total number of rotations for each rat was close to zero. Each rat was tested two times, once with 30 Hz DBS and once without DBS in a counterbalanced way with a washout period of 48 h.

##### Effect of IC glutamatergic manipulations

**Figure 1.**
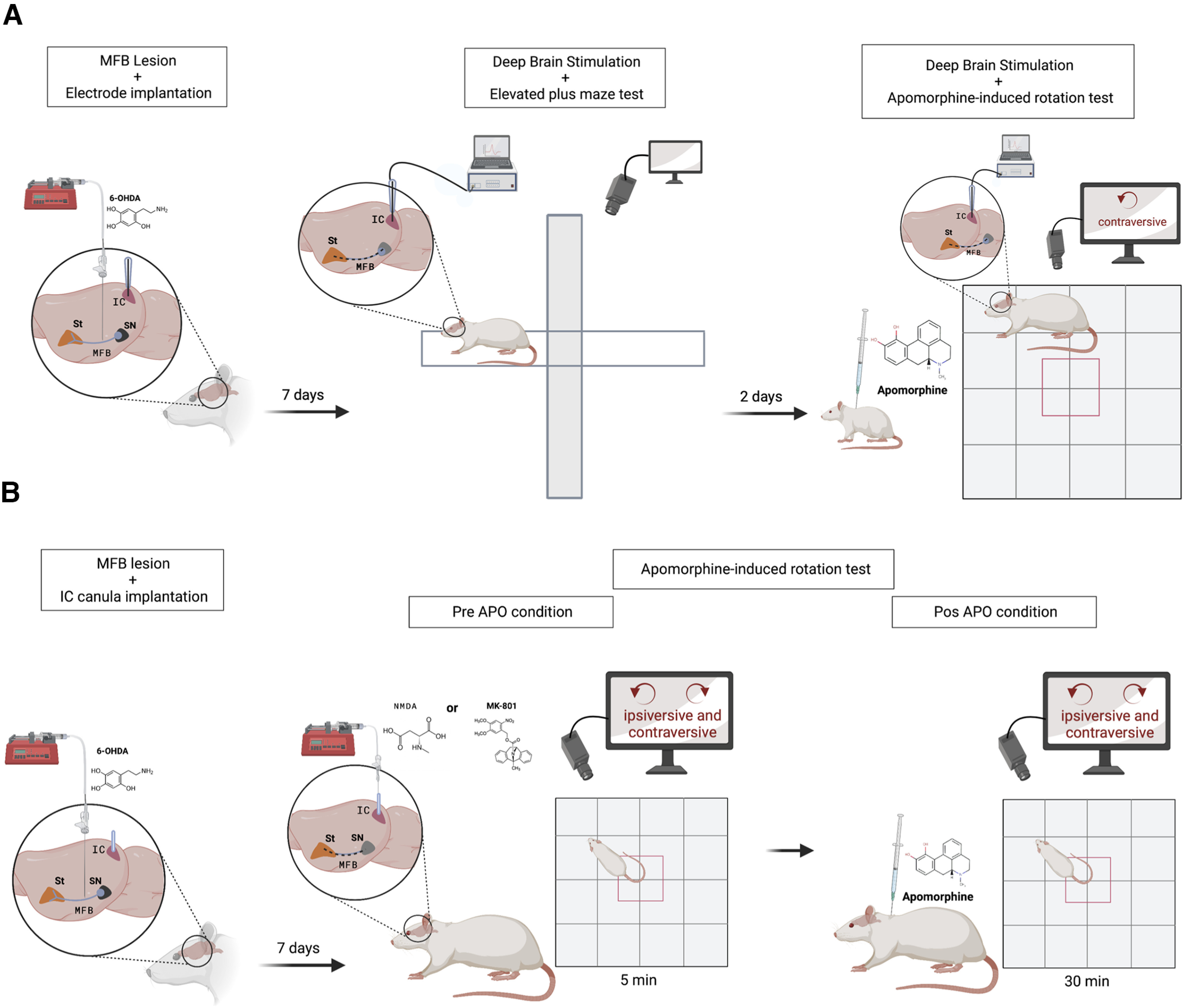
Graphical representation summarizing the behavioral procedures. ***A***, Assessment of IC 30 Hz DBS on behavior in the EPM and on apomorphine-induced rotations in hemiparkinsonian rats. Rats were placed on the EPM under 30 Hz DBS a week after 6-OHDA-produced unilateral lesions in the MFB. Two days later, the same rats received apomorphine and the rotations were assessed under 30 Hz DBS. ***B***, Assessment of the role of IC glutamate on apomorphine-induced rotations in hemiparkinsonian rats. Rotations were recorded during 5 min immediately after IC microinjection of NMDA or MK-801 (pre-APO condition). Then, the same rats received apomorphine subcutaneously, and rotations were again recorded over 30 min (post-APO condition). St, Neostriatum (created with BioRender.com).

The rats that received guide cannulas implanted in the IC were randomly assigned to the following groups: Lesion (*n* = 26); and Sham lesion (*n* = 18). A given microinjection was delivered using a stainless steel dental needle (30 gauge; Miraject) introduced through the guide cannula previously implanted in the IC, until its lower end was 1 mm below the cannula tip. This dental needle (length, 14 mm) was connected by a polyethylene tube to a 10 ml Hamilton syringe, and the drugs were delivered at a rate of 0.5 μl/min controlled by a microinjection pump (model sp101i, WPI). The animals from the control groups received an equivalent volume of physiological saline. The dental needle was left in place for an additional 1 min thereafter. Immediately after receiving an injection of NMDA (unilateral), MK-801 (unilateral or bilateral), or a physiological saline solution into the IC, the rats were placed individually into the arena and their behavior was recorded over 5 min (pre-APO condition). Then, APO was injected subcutaneously and the behavior was recorded over 30 min (post-APO condition). The number of whole rotations (360° turns) toward either the side of the lesion (ipsiversive) or the opposite side (contraversive) was assessed offline by a blind to the protocol experimenter. Only the number of narrow rotations (within a diameter of <30 cm corresponding to the common description “head-to-tail”) were considered and two half-rotations were counted as one whole rotation. The rats were tested two times with a washout period of 48 h [Fig. 1*A*,*B* (created with BioRender.com)].

### Assessment of 6-OHDA lesion and electrode location in the IC

At the end of the behavioral experiments, each rat received an overdose of sodium-pentobarbital (Fagron; 600 mg/kg). When breathing stopped, the same stimulation cable used during the behavioral experiments was connected to the working electrode and the electrical stimulation (current intensity, 50 μA; pulse width, 100 μs; pulse interval, 100 μs) was applied for 90 s to produce a small local electrolytic lesion. This step was crucial to better localize electrode tip placement during the later histologic analysis afterward. The rats were then perfused intracardially with 4% paraformaldehyde. Thirty-micrometer-thick coronal sections were cut with a cryostat (model 1850, Leica) and mounted on slides. Histologic staining for tyrosine hydroxylase (TH; Graybiel et al., 1987) was used to assess the loss of DA cells in the SNpc ipsilateral and contralateral to the 6-OHDA injection. The rats were aleatorily assigned into No-DBS and DBS groups. Only rats presenting a marked degeneration of the DAergic neurons in the SNpc with >80% of neuronal loss were included in the present study. Sections with the IC were stained with cresyl violet to reveal the stimulation sites. Boundaries of the SN and IC were assessed with reference to the atlas of Paxinos and Watson (2007).

### Electrophysiological assessment

The goal of this experiment was to investigate whether the intracollicular glutamatergic manipulations may affect neural activity in the neostriatum. For that, intrastriatal neural activity was assessed after microinjection of either a glutamatergic agonist (NMDA) or antagonist (MK-801) into the IC in anesthetized rats. For an overview of this study, see Figure 5*A* (created with BioRender.com).

#### Equipment

The microdrive and microelectrodes used in this experiment were described in detail by Eckhorn and Thomas (1993). Briefly, the microdrive (Mini Matrix, Thomas RECORDING), fixed to the stereotaxic equipment, carries up to five stainless steel tubes (outside diameter, 305 μm; inside diameter, 152 μm). In our experiment, one microelectrode (impedance range, 0.3–2.3 MΩ) was placed inside each tube with the tip of each microelectrode ∼1 mm back from the distal end of the tube. Four electrodes were used for multiunit recording (platinum/tungsten alloy; impedance, 1–2 MΩ). Each microelectrode was connected to a high-impedance headstage preamplifier integrated into the Mini Matrix microdrive. The preamplifier was connected to a mode selection and impedance test control device that allowed the selection of one of three functions for each microelectrode, as follows: neural recording, impedance testing, and electrical stimulation. The recorded signals were then preamplified (gain = 19; Mini Matrix), main-amplified (gain = 500; MAF-05, Thomas RECORDING), and filtered (bandpass, 500 Hz to 20 kHz), then passed through a data acquisition system and digitized. Neural activity was displayed and archived using the MC-Rack program (Multichannel Systems).

#### Electrophysiological recordings

Rats were anesthetized with urethane (1.4 g/kg, i.p.; Sigma-Aldrich) and fixed in a stereotaxic frame (David Kopf Instruments) with the upper incisor bar set at 3.3 mm below the interaural line such that the skull was horizontal between bregma and λ. A midline scalp incision was made, and a 2 mm burr hole was drilled in the skull above the IC. A dental needle connected to a Hamilton syringe was introduced vertically into the IC for drug microinjections. Another hole (∼5 mm) was opened in the skull above the neostriatum. The Mini Matrix was lowered until the tip of each tube rested just over the dural surface with the electrode tips at the same depth and aligned in parallel at a distance of 305 μm from each other. Then, the electrodes were moved into the brain aiming at the neostriatum according to the following coordinates using bregma as the reference: AP, 1.4 mm; ML, ±2.5 mm; DV, 4.5 mm. Under computer control (Motor Control Software, Thomas RECORDING), each microelectrode was moved independently at a speed of 20 μm/s crossing the dura mater toward the neostriatum. Once in the neostriatum, recordings were made from two different depths (4.5 and 6.5 mm from the dura mater were considered as more dorsal and more ventral regions of the neostriatum, respectively) and were considered as baseline measurements. Then, either NMDA, MK-801, or physiological saline was microinjected into the IC. One minute after removal of the microinjection needle, intrastriatal neural recordings from the same depths used for baseline measurements were performed. At the end of the experiments, an iron electrode coated with quartz (outside diameter, 80 m) was introduced into the brain, through the fifth stainless steel tube, at the same depth as the recording electrodes. An electrical current of 25 μA was applied twice (30 and 90 s) to deposit iron near the prior recording sites. Experimental sessions lasted no more than 2 h, and each animal received only one IC microinjection. The level of anesthesia was monitored by frequently checking the response to tail pinch, and urethane was supplemented as necessary to maintain the depth of anesthesia.

### Neural tract tracing of pathways connecting the IC

An independent group of intact Wistar rats (*n* = 4) was anesthetized intraperitoneally with a mixture of ketamine (92 mg/kg; União Química Farmacêutica Nacional) and xylazine (9.2 mg/kg; Hertape/Calier). They were fixed in a stereotaxic frame (David Kopf Instruments), and the bidirectional neural tract tracer biotinylated dextran amine [BDA; 3.000 molecular weight (MW); Thermo Fisher Scientific] was microinjected into the IC through a microinjection cannula (Mizzy; 30 gauge; outer diameter, 0.3 mm, length 14 mm; see Fig. 6*A*,*B*) following the coordinates described for item 2.3. BDA was deposited in the IC at a volume of 0.5 μl over the course of 5 min. Infusions were delivered using an infusion pump (Stoelting) through a polyethylene tube (PE10) attached to the cannula.

Seven days after microinjection, the rats were again anesthetized with ketamine and xylazine, and perfused through the left cardiac ventricle with cold, oxygen-enriched, Ca^2+^-free Tyrode’s buffer (40 ml at 4°C) and ice-cold paraformaldehyde (200 ml, 4% (w/v) in 0.1 m sodium phosphate buffer, pH 7.3) for 15 min at a pressure of 50 mmHg with a perfusion pump (L/S peristaltic tubing pump, Masterflex). The brainstem was quickly removed, sectioned, and immersed in fresh fixative for 4 h at 4°C. It was then rinsed for at least 12 h each in 10% and 20% sucrose dissolved in 0.1 m sodium phosphate buffer, pH 7.4, at 4°C. The tissue pieces were immersed in 2-methylbutane (Sigma-Aldrich), frozen on dry ice, embedded in Tissue-Tek OCT, and cut with a cryostat (model CM 1950 Leica) at −22°C. The 20 μm slices were subsequently mounted on glass slides (coated with chrome alum-gelatin to prevent detachment) and stained with hematoxylin-eosin using an Autostainer (CV 5030 Autostainer XL, Leica). The immunohistochemistry was based on previous studies (see for details Almada et al., 2015, 2021; Falconi-Sobrinho et al., 2017). The positions of the guide cannula tips were determined according to the atlas of Paxinos and Watson (2007) under a motorized photomicroscope (AxioImager Z1, Zeiss).

### Statistical analysis

The data obtained in the EPM test were analyzed using a three-way ANOVA for the factors lesion, DBS, and location (open × closed arms) or lateralization (ipsiversive × contraversive) to detect significant differences between groups. Bonferroni’s *post hoc* pairwise tests were used to determine the sources of detected significances. Repeated-measures two-way ANOVA, followed by Bonferroni’s *post hoc* test when appropriate, was performed to compare the total number of risk assessments, turns, half turns, and rotations. The effect of DBS during APO-induced rotation behavior was analyzed using repeated-measures two-way ANOVA with the following factors: lesion and DBS, electrode placement and lesion or electrode placement, and DBS. ANOVA was followed by Bonferroni’s *post hoc* test when appropriate. Additional two-way ANOVAs were conducted for comparing the effect of microinjections at sham lesion and lesion conditions, with treatment (NMDA, MK-801, or saline) and rotations (ipsiversive or contraversive) factor followed by Bonferroni’s *post hoc* test when appropriate.

The number of TH-labeled cells in both the lesion and no-lesion sides in the SNpc of rats receiving 30 Hz DBS or IC microinjections was compared by one-way ANOVA, followed by either Tukey’s *post hoc* tests or unpaired Student’s *t* test, respectively. Statistical analyses were performed using GraphPad Prism version 9.0. All behavioral data were expressed as the mean ± SEM. Differences were considered significant when the *p* value was <0.05.

For assessment of neural activity in the neostriatum, and in a first step, raw multiunit activity was analyzed offline with the Thomas Spike Sorter Software (Thomas RECORDING), using nonlinear energy operator and principal component analysis to detect single unit activity and classify spikes. Afterward, spike times were analyzed further using MATLAB 2015a (MathWorks) and converted to spike density functions using a Gaussian smoothing window of 90 ms width (σ = 30 ms) to obtain continuous firing rates of each single unit. Finally, single-unit activity for each animal and condition was averaged across time, and all neurons for each specific depth. These averaged firing rates were compared with respect to recording depth and/or injected drug by using two-sample Student’s *t* tests without assuming equal variances. Furthermore, it was analyzed the mean activity of neurons in the neostriatum. This was done either before a microinjection (baseline measurement) or right after a microinjection with NMDA, MK-801, or physiological saline at two different penetration depths.

## Results

An overview of the behavioral procedures can be seen in Figure 1, *A* and *B* (created with BioRender.com). Briefly, 1 week after receiving a unilateral microinjection of 6-OHDA in the MFB and having an electrode implanted in the right or left IC, the rats were placed on an EPM receiving intracollicular DBS to assess spontaneous lateralization (for rationale, see Materials and Methods). Two days later, these rats were assessed for DBS effects on APO-induced contraversive rotations in an open field. Another group of rats received a microinjection of 6-OHDA in the MFB, and a microinjection cannula was implanted bilaterally in the IC. One week later, the glutamatergic antagonist MK-801 or the agonist NMDA was microinjected in the IC and spontaneous (pre-APO condition) or APO-induced rotations (post-APO condition) were assessed in the open field.

### Behavioral assessment during the EPM test

**Figure 2.**
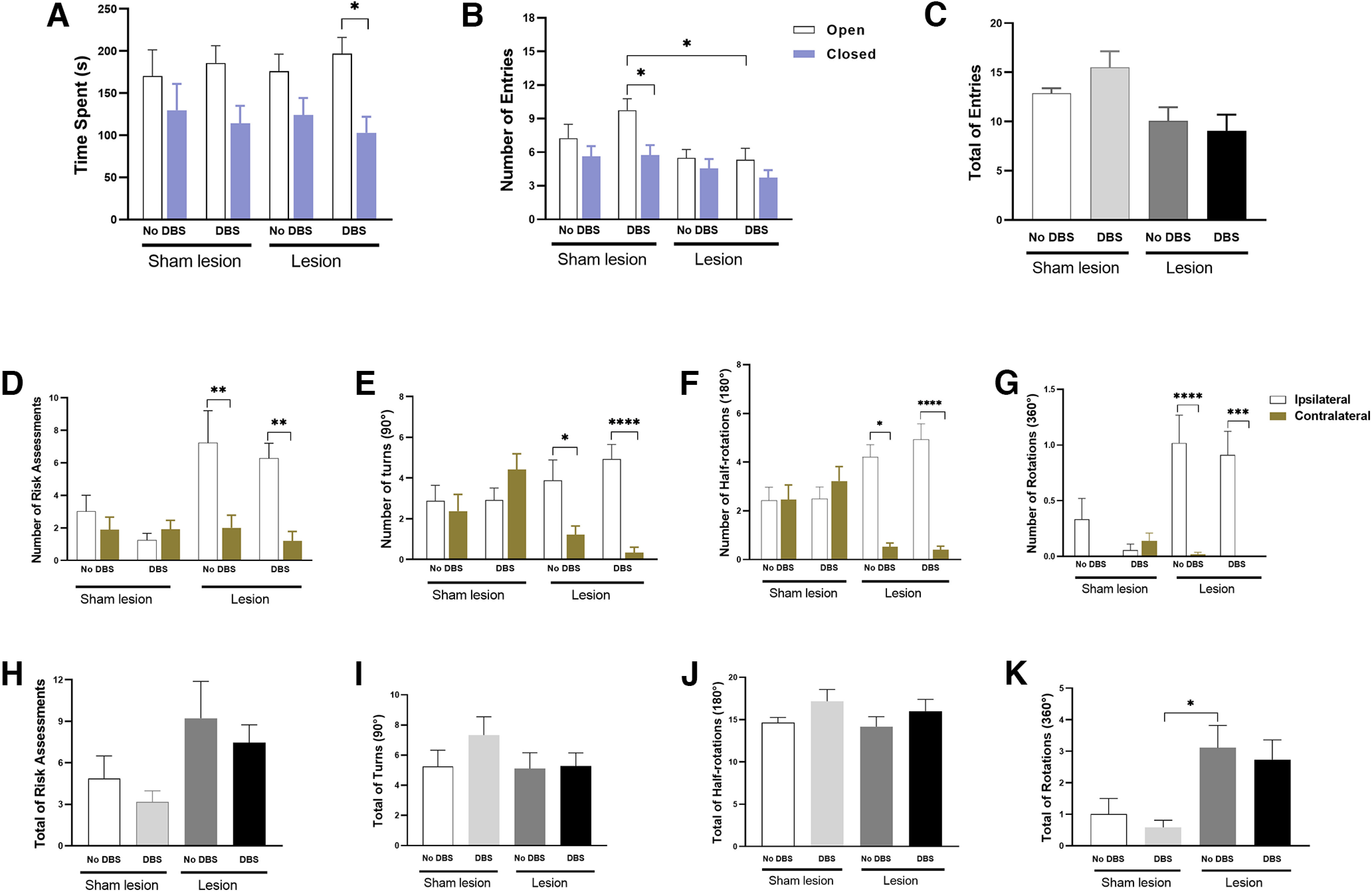
Effect of intracollicular 30 Hz DBS on exploratory behavior of hemiparkinsonian rats placed on the EPM test. ***A–C***, Classic behaviors usually assessed during the EPM test, such as time spent (***A***), number of entries in the open and closed arms (***B***), and total number of entries (***C***), were recorded from 6-OHDA and Sham control rats. Since the test was conducted under lower illumination, no anxiety was observed and the rats were encouraged to explore the arms more, allowing the experimenter to investigate whether the exploratory behavior was biased to either side. ***D–G***, 6-OHDA rats showed an increased number of risk assessments (***D***), turns (***E***), half-rotations (***F***), and rotations toward the lesioned side (ipsilateral; ***G***). Thirty hertz DBS did not affect this lateralization. ***H–K***, There was no difference in the total number of these exploratory behaviors when comparing Lesion and Sham lesion groups. Bars represent mean values, and vertical lines indicate SEM values. *p < 0.05; **p < 0.01; ***p < 0.001; ****p < 0.0001, according to ANOVA followed by Bonferroni’s multiple-comparisons *post hoc* test.

Figure 2*A* shows the time spent in the arms for all groups. According to a three-way ANOVA, there was a main effect of location (*F*_(1,62)_ = 14.21, *p* < 0.001), but no effect of lesion (*F*_(1,36)_ = 0.000, *p* > 0.05) or DBS treatment (*F*_(1,62)_ = 0.000, *p* > 0.05) and no interaction lesion × DBS (*F*_(1,36)_ = 0.000, *p* > 0.05) or DBS × location (*F*_(1,62)_ = 1.268, *p* > 0.05), lesion × location (*F*_(1,36)_ = 0.3648, *p* > 0.05), or DBS × lesion × location (*F*_(1,36)_ = 0.0173, *p* > 0.05). Bonferroni’s *post hoc* comparisons indicated an effect of location only in the DBS lesion group, since these rats when receiving 30 Hz DBS spent more time in the open arms (*p* < 0.05). Importantly, the lesion No-DBS group and both Sham lesion groups revealed no preference for any arms (*p* > 0.05), suggesting that, as expected, the EPM test conditions were not anxiogenic.

Regarding the number of entries, a three-way ANOVA revealed an effect of lesion (*F*_(1,98)_ = 11.74, *p* < 0.001) and location (*F*_(1,98)_ = 9.151, *p* < 0.01) but no effect of DBS treatment (*F*_(1,98)_ = 0.3670, *p* > 0.05; Fig. 2*B*), interaction DBS × lesion (*F*_(1,98)_ = 1.791, *p* > 0.05), DBS × location (*F*_(1,98)_ = 1.259, *p* > 0.05), lesion × location (*F*_(1,98)_ = 1.301, *p* > 0.05), or DBS × lesion × location (*F*_(1,98)_ = 0.4054, *p* > 0.05). When comparing the total number of entries, a two-way ANOVA showed a significant effect of lesion (*F*_(1,49)_ = 8.253, *p* < 0.01) but no effect of DBS (*F*_(1,49)_ = 0.2580, *p* > 0.05) or lesion × DBS interaction (*F*_(1,49)_ = 1.259, *p* > 0.05; Fig. 2*C*). According to Bonferroni’s *post hoc* comparisons, rats with sham lesions and DBS entered more often into the open arms compared with the closed arms (*p* < 0.05) and compared with the number of open arm entries of 6-OHDA rats receiving DBS (*p* < 0.05).

Regarding the number of risk assessments, a three-way ANOVA revealed an effect of lesion (*F*_(1,98)_ = 6.184, *p* < 0.05) and lateralization (*F*_(1,98)_ = 9.552, *p* < 0.01) but no effect of DBS treatment (*F*_(1,98)_ = 0.9923, *p* > 0.05). There was a significant lesion × lateralization interaction (*F*_(1,98)_ = 7.992, *p* < 0.01; Fig. 2*D*) but no DBS × lesion (*F*_(1,98)_ = 0.0001844, *p* > 0.05), DBS × lateralization (*F*_(1,98)_ = 0.3136, *p* > 0.05), or DBS × lesion × lateralization (*F*_(1,98)_ = 0.2214, *p* > 0.05) interaction. Bonferroni’s *post hoc* comparisons revealed that 6-OHDA rats showed more ipsilateral risk assessments (*p* < 0.01) and this lateralization was not affected by DBS treatment (*p* > 0.05). A two-way ANOVA revealed that the total number of risk assessments was not affected by lesion or 30 Hz DBS treatment (*F*_(3,49)_ = 1.785, *p* > 0.05; Fig. 2*H*).

Figure 2*E–G* shows the results for rotatory behaviors. Regarding the number of turns, according to a three-way ANOVA, there was a significant effect of lateralization (*F*_(1,98)_ = 8.539, *p* < 0.01) and lesion × lateralization interaction (*F*_(1,98)_ = 14.86, *p* < 0.001). There was no effect of DBS (*F*_(1,98)_ = 1.090, *p* > 0.05), lesion (*F*_(1,98)_ = 1.058, *p* > 0.05), or DBS × lesion (*F*_(1,98)_ = 0.8081, *p* > 0.05), DBS × lateralization (*F*_(1,98)_ = 0.0009664, *p* > 0.05), or DBS × lesion × lateralization (*F*_(1,98)_ = 3.364, *p* > 0.05) interaction. *Post hoc* Bonferroni’s multiple comparisons test revealed that 6-OHDA rats showed more ipsilateral turns (No-DBS group, *p* < 0.05; DBS group, *p* < 0.0001; Fig. 2*E*).

Regarding the number of half rotations, according to a three-way ANOVA, there was a significant effect of lateralization (*F*_(1,98)_ = 48.63, *p* < 0.0001) and lesion × lateralization interaction (*F*_(1,98)_ = 70.60, *p* < 0.0001). There was no effect of DBS (*F*_(1,98)_ = 1.861, *p* > 0.05), lesion (*F*_(1,98)_ = 0.2568, *p* > 0.05), and DBS × lesion (*F*_(1,98)_ = 0.04,878, *p* > 0.05), DBS × lateralization (*F*_(1,98)_ = 0.02,458, *p* > 0.05), or DBS × lesion × lateralization (*F*_(1,98)_ = 2.045, *p* > 0.05) interaction. *Post hoc* Bonferroni’s multiple-comparisons test revealed that 6-OHDA rats showed more ipsilateral turns (No-DBS group, *p* < 0.05; DBS group, *p* < 0.0001; Fig. 2*F*).

Regarding the number of full rotations, according to a three-way ANOVA, there was a significant effect of lateralization (*F*_(1,98)_ = 23.66, *p* < 0.0001) and lesion (*F*_(1,98)_ = 10.22, *p* < 0.0001), but no effect of DBS (*F*_(1,98)_ = 0.3552, *p* > 0.05). There was a significant lesion × lateralization interaction (*F*_(1,98)_ = 13.98, *p* < 0.001) but no DBS × lesion (*F*_(1,98)_ = 0.0008511, *p* > 0.05), DBS × lateralization (*F*_(1,98)_ = 1.295, *p* > 0.05), or DBS × lesion × lateralization (*F*_(1,98)_ = 0.5442, *p* > 0.05) interaction. *Post hoc* Bonferroni’s multiple-comparisons test revealed that 6-OHDA rats showed more ipsilateral rotations and that DBS treatment did not affect this asymmetry (No-DBS group, *p* < 0.0001; DBS group, *p* < 0.001; Fig. 2*G*).

According to a two-way ANOVA, there were no significant effects in the total number of turns (*F*_(3,49)_ = 0.9136, *p* > 0.05; Fig. 2*I*), or in the total number of half-rotations (*F*_(3,49)_ = 1.113, *p* > 0.05; Fig. 2*J*). There was a significant difference in the total number of rotations (*F*_(3,49)_ = 3.911, *p* < 0.05; Fig. 2*K*) with the lesion No-DBS group showing more rotations than the Sham lesion DBS group (*p* < 0.05).

An additional analysis was performed considering the placement of the IC electrode regarding the lesion side (ipsilateral or contralateral) as a factor. A three-way ANOVA used to analyze the time spent revealed an effect of location (open and closed arms; *F*_(1,29)_ = 6.139, *p* < 0.05) but no effect of DBS (*F*_(1,29)_ = 0.000, *p* > 0.05), electrode placement (*F*_(1,29)_ = 0.000, *p* > 0.05), or location × DBS (*F*_(1,29)_ = 0.5090, *p* > 0.05), electrode placement × DBS (*F*_(1,29)_ = 0.000, *p* > 0.05), location × electrode placement (*F*_(1,29)_ = 0.05,331, *p* > 0.05), or DBS × location × electrode placement (*F*_(1,29)_ = 0.05958, *p* > 0.05) interaction. Regarding the number of entries, according to a three-way ANOVA, there was a significant effect of location (open and closed arms; *F*_(1,29)_ = 6.078, *p* < 0.05) but no effect of DBS (*F*_(1,29)_ = 0.2191, *p* > 0.05), electrode placement (*F*_(1,29)_ = 0.4162, *p* > 0.05), or location × DBS (*F*_(1,29)_ = 0.3384, *p* > 0.05), electrode placement × DBS (*F*_(1,29)_ = 0.6761, *p* > 0.05), location × electrode placement (*F*_(1,29)_ = 0.0069, *p* > 0.05), or DBS × location × electrode placement (*F*_(1,29)_ = 0.4059, *p* > 0.05) interaction. Regarding the number of risk assessments, three-way ANOVAs revealed a significant effect of lateralization (*F*_(1,29)_ = 33.33, *p* < 0.0001) but no effect of DBS (*F*_(1,29)_ = 0.1439, *p* > 0.05) electrode placement (*F*_(1,29)_ = 0.7433, *p* > 0.05), or DBS × lateralization (*F*_(1,29)_ = 0.000, *p* > 0.05), DBS × electrode placement (*F*_(1,29)_ = 0.6292, *p* > 0.05), lateralization × electrode placement (*F*_(1,29)_ = 0.3772, *p* > 0.05), or DBS × lateralization × electrode placement (*F*_(1,29)_ = 0.06,035, *p* > 0.05) interaction. Regarding the number of turns, three-way ANOVA revealed a significant effect of lateralization (*F*_(1,29)_ = 28.91, *p* < 0.0001) but no effect of DBS (*F*_(1,29)_ = 0.02,968, *p* > 0.05), electrode placement (*F*_(1,29)_ = 0.7419, *p* > 0.05), or DBS × lateralization (*F*_(1,29)_ = 2.281, *p* > 0.05), DBS × electrode placement (*F*_(1,29)_ = 0.04,433, *p* > 0.05), lateralization × electrode placement (*F*_(1,29)_ = 1.609, *p* > 0.05), or DBS × lateralization × electrode placement (*F*_(1,29)_ = 0.03,284, *p* > 0.05) interaction. Regarding the number of half-rotations, three-way ANOVA revealed a significant effect of lateralization (*F*_(1,29)_ = 86.52, *p* < 0.0001) but no effect of DBS (*F*_(1,29)_ = 1.704 *p* > 0.05), electrode placement (*F*_(1,29)_ = 0.1336, *p* > 0.05), or DBS × lateralization (*F*_(1,29)_ = 2.129, *p* > 0.05), DBS × electrode placement (*F*_(1,29)_ = 0.1336, *p* > 0.05), lateralization × electrode placement (*F*_(1,29)_ = 1.955, *p* > 0.05), or DBS × lateralization × electrode placement (*F*_(1,29)_ = 0.1331, *p* > 0.05) interaction. Regarding the number of rotations, a three-way ANOVA revealed a significant effect of lateralization (*F*_(1,29)_ = 27.66, *p* < 0.0001) but no effect of DBS (*F*_(1,29)_ = 0.3208, *p* > 0.05), electrode placement (*F*_(1,29)_ = 0.2.363, *p* > 0.05), or DBS × lateralization (*F*_(1,29)_ = 32.08, *p* > 0.05), DBS × electrode placement (*F*_(1,29)_ = 0.1637, *p* > 0.05), lateralization × electrode placement (*F*_(1,29)_ = 2.363, *p* > 0.05), or DBS × lateralization × electrode placement (*F*_(1,29)_ = 0.1637, *p* > 0.05; data not shown) interaction. These results suggest that lesion-induced spontaneous lateralization was not affected by DBS regardless of whether the electrodes were placed ipsilateral or contralateral to the lesion.

### Assessment of APO-induced rotation during 30 Hz DBS

**Figure 3.**
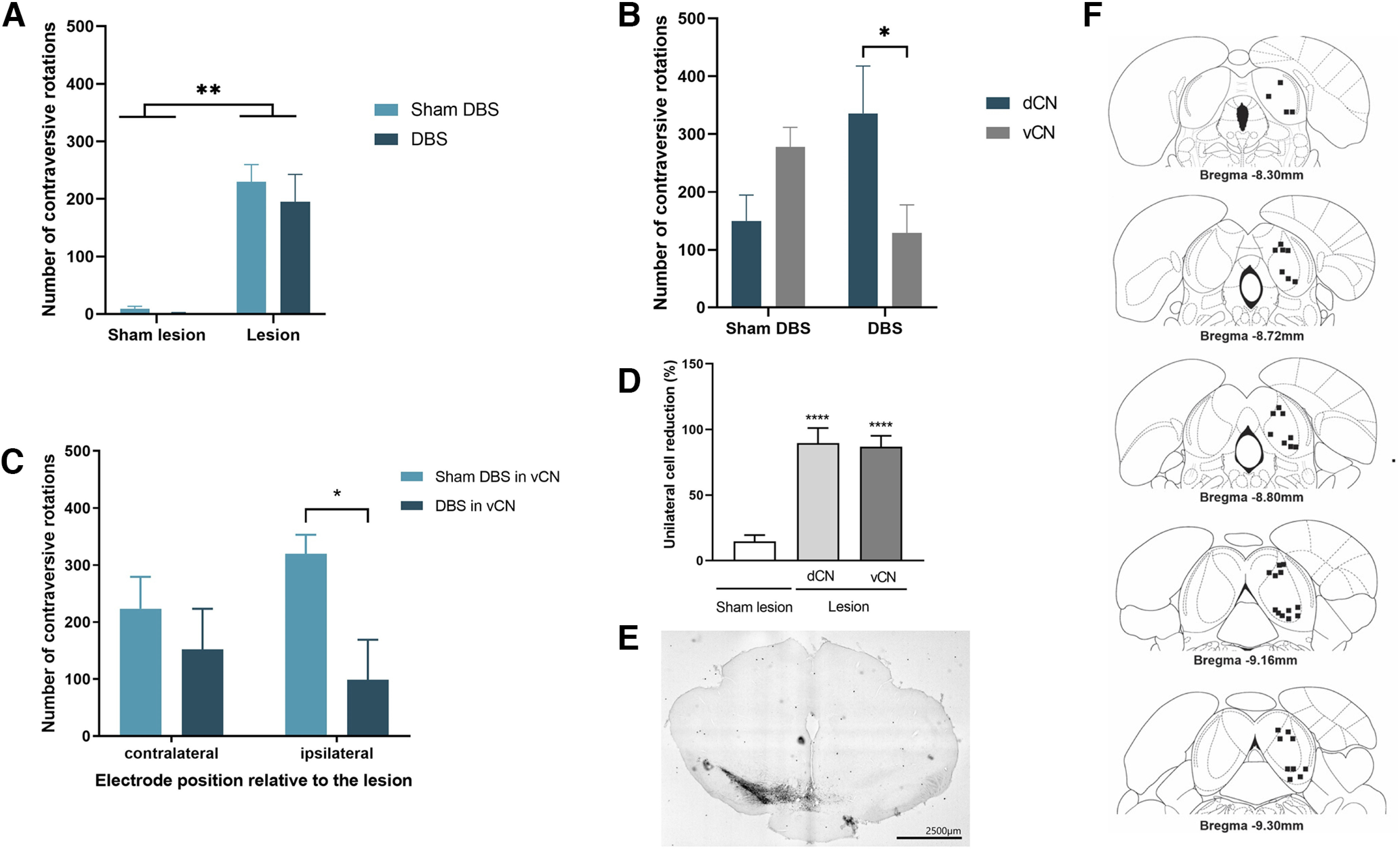
Intracollicular 30 Hz DBS reduces APO-induced contraversive rotations of hemiparkinsonian rats. ***A***, Only the 6-OHDA groups displayed a significant rotational bias toward the opposite side of the lesion. ***B***, When stimulated in the vCN, but not in the dCN, of the IC, these rats rotated less during 30 Hz DBS. ***C***, When the electrode was implanted in the vCN of the IC ipsilateral, but not contralateral, to the lesion side, 30 Hz DBS reduced significantly contraversive rotations. ***D***, ***E***, The presence of fewer TH-labeled cells in the SNpc for Sham lesion rats compared with 6-OHDA rats with electrodes in the dCN or vCN of the IC (***D***) is demonstrated by the percentage of cell reduction and by a representative image comparing both the lesion and intact side at the SN (***E***). ***F***, The locations of electrode tips (black points) in cross-section diagrams of the IC are shown according to the atlas of Paxinos and Watson (2007). The number of points is less than the total of animals studied because of graphical overlaps. Bars represent mean values, and vertical lines indicate SEM values. *p < 0.05; **p < 0.01; ****p < 0.0001, according to ANOVA, followed by Bonferroni’s multiple-comparisons *post hoc* test.

A two-way ANOVA for repeated measures of APO-induced rotation revealed a significant effect of lesion (*F*_(1,23)_ = 29.60, *p*< 0.0001) but no effect of DBS (*F*_(1,23)_ = 0.1568, *p*> 0.05) or lesion × DBS interaction (*F*_(1,23)_ = 0.06,935, *p* > 0.05). *Post hoc* Bonferroni’s multiple-comparisons test demonstrated that only the lesion group displayed a significant rotational bias toward the opposite side of the lesion (contraversive rotations) compared with Sham lesion group (*p* < 0.01; Fig. 3*A*). However, a new analysis was performed after the identification of electrode tip positioning. Rats with electrode tips at the dCN and vCN of the IC were separated into two different subgroups. When considering the intracollicular site of electrode placement, a relevant factor, two-way ANOVA, revealed a significant DBS × electrode placement interaction (*F*_(1,32)_ = 9.840, *p* < 0.01). Bonferroni’s *post hoc* comparisons demonstrated that rats stimulated in the vCN rotated less compared with dCN or Sham DBS groups (*p* < 0.05; Fig. 3*B*). An additional two-way ANOVA was performed to analyze the effect of DBS applied in the vCN, considering the electrode position related to lesion side (ipsilateral or contralateral) as a factor. The two-way ANOVA revealed a significant effect of DBS (*F*_(1,10)_ = 6.663, *p* < 0.05) but no electrode position × DBS interaction (*F*_(1,10)_ = 1.752, *p* > 0.05). According to Bonferroni’s *post hoc* test, rats submitted to vCN intracollicular DBS applied ipsilaterally to the side of the lesion displayed significantly less contraversive rotations (*p* < 0.05) when compared with No-DBS in the vCN group. When 30 Hz DBS was applied in the vCN contralateral to the lesion side, the number of contraversive rotations was not significantly different from those displayed by rats from the No-DBS in the vCN group (*p* > 0.05; Fig. 3*C*).

### Assessment of spontaneous rotation after either MK-801 or NMDA microinjections in the IC

**Figure 4.**
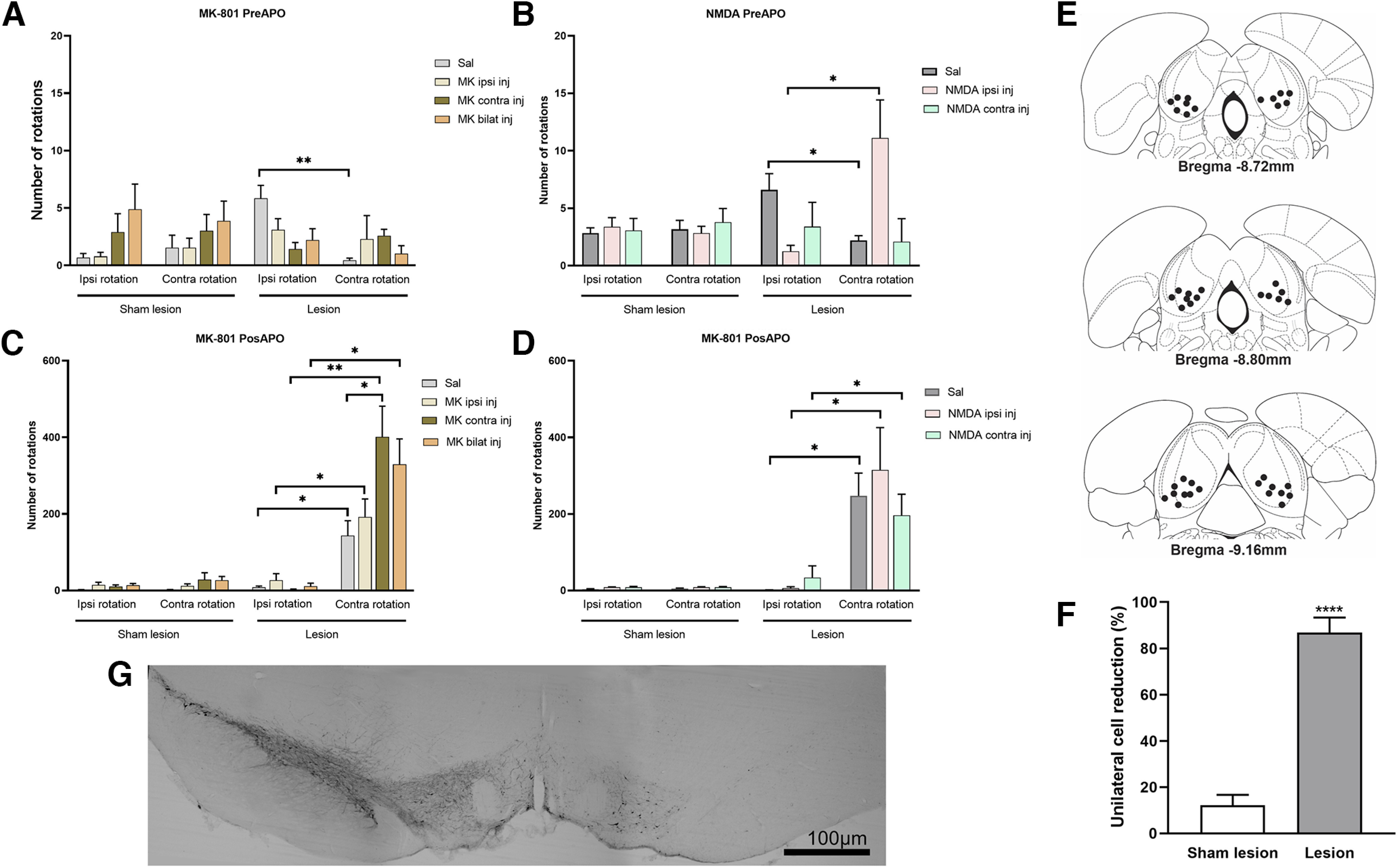
Effect of MK-801 or NMDA microinjected into the IC of hemiparkinsonian rats on spontaneous rotations (pre-APO condition, top panel) and APO-induced rotations (post-APO condition, bottom panel). ***A***, Lesion rats receiving physiological saline showed a clear ipsiversive asymmetry, which disappeared after MK-801 intracollicular microinjection, regardless of the microinjection side (ipsilaterally, contralaterally, or bilaterally) in the IC. No asymmetry was displayed by sham control rats treated with either microinjections of physiological saline or MK-801 in the IC. ***B***, When treated with physiological saline in the IC, 6-OHDA rats exhibited an ipsiversive asymmetry. NMDA microinjected in the IC ipsilateral to the lesion reverted the lateralization to a clear contraversive asymmetry. No asymmetry was observed after NMDA microinjection in the IC contralateral to the lesion. There was no effect of IC treatment nor any asymmetry in rats with sham lesions. ***C***, After receiving APO subcutaneously, 6-OHDA rats exhibited a clear contraversive asymmetry. This asymmetry was significantly potentiated by MK-801 microinjected in the IC contralateral to the lesion side. The sham groups that received either MK-801 or physiological saline in the IC did not exhibit any asymmetry. ***D***, 6-OHDA rats exhibited the highest number of contraversive rotations, indicating a clear asymmetry as a consequence of subcutaneous APO treatment. Intracollicular NMDA treatment did not affect that asymmetry regardless of the side of IC microinjection with respect to the lesion. Microinjection of either physiological saline or NMDA in the IC of sham rats did not induce any asymmetry during rotations. ***E***, Location of microinjections sites in cross-section diagrams of the IC, according to the atlas of Paxinos and Watson (2007). The number of points is less than the total of animals studied because of graphical overlaps. ***F***, ***G***, Fewer TH-labeled cells in the SNpc for Lesion group compared with the Sham lesion group is demonstrated by the percentage of cell reduction (***F***) and by a representative image comparing intact and lesion sides of the same rat (***G***). Bars represent mean values, and vertical lines indicate SEM values. *p < 0.05; **p < 0.01, according to ANOVA, followed by Bonferroni’s multiple-comparisons *post hoc* test.

The effect of MK-801 microinjected into the IC on spontaneous rotation (before apomorphine; injection; Fig. 4). Two independent two-way ANOVAs were performed for sham lesion and lesion groups. Regarding the sham lesion groups, a two-way ANOVA for repeated measures revealed no effect of rotations (*F*_(1.16)_ = 0.03,625; *p* > 0.05), treatment (*F*_(1.903,30.45)_ = 2.877; *p* > 0.05), or behavior × treatment interaction (*F*_(3.48)_ = 0.2183; *p* > 0.05), and therefore no asymmetry was observed. Regarding the lesion groups, a two-way ANOVA for repeated measures revealed a significant effect of behavior (*F*_(1.118)_ = 5.020; *p* < 0.05) and of behavior × treatment interaction (*F*_(3.118)_ = 3.989; *p* < 0.01), but no effect of treatment (*F*_(2.014,79.20)_ = 0.9569; *p* > 0.05). When treated with physiological saline, 6-OHDA rats rotated spontaneously more toward the lesion side (*post hoc* Bonferroni’s multiple-comparisons test: ipsiversive rotations, *p* < 0.001; Fig. 4*A*), revealing a clear ipsiversive asymmetry. This asymmetry disappeared after MK-801 microinjection (i.e., there was no difference when comparing the number of ipsiversive and contraversive rotations; *p* > 0.05) performed by lesioned rats that received either ipsilateral or contralateral, or even bilateral, MK-801 microinjections into the IC.

Figure 4*B* shows the effect of NMDA microinjected into the IC on spontaneous rotation behavior in 6-OHDA rats. Two independent two-way ANOVAs were performed for Sham lesion and Lesion groups. Regarding the Sham lesion groups, a two-way ANOVA for repeated measures revealed no effect of behavior (*F*_(1.16)_ = 0.06,336; *p* > 0.05), treatment (*F*_(1.536,24.575)_ = 0.1233; *p* > 0.05) or behavior × treatment interaction (*F*_(2,32)_ = 0.2841; *p* > 0.05; i.e., there was no significant effect of IC treatment and no asymmetry was observed; *p* > 0.05). Regarding the lesion groups, a repeated-measures two-way ANOVA revealed a significant behavior × treatment interaction (*F*_(2,36)_ = 9.512; *p* < 0.001), but no effect of behavior (*F*_(1,18)_ = 0.5725; *p* > 0.05) or treatment (*F*_(1.822,32.80)_ = 1.988; *p* > 0.05). When treated with physiological saline in the IC, 6-OHDA rats performed more ipsiversive than contraversive rotations (Bonferroni’s multiple-comparisons test; *p* < 0.05; i.e., they exhibited an ipsiversive asymmetry. Notably, there was a significant increase in the number of contraversive rotations after NMDA microinjection into the IC ipsilateral to the lesion (*p* < 0.05), indicating a clear contraversive asymmetry induced by NMDA treatment. In addition, NMDA microinjected into the IC contralateral to the lesion abolished lateralization, since there was no significant difference when comparing the number of ipsiversive and contraversive rotations (*p* > 0.05).

### Assessment of the rotations induced by APO after either MK-801 or NMDA microinjections into the IC

When analyzing the number and direction of rotations induced by APO after intracollicular MK-801 microinjection from Sham lesion groups, a two-way ANOVA for repeated measures revealed no effect of treatment (*F*_(1.693,27.09)_ = 20.49; *p* > 0.05), rotations (*F*_(1,16)_ = 1.164; *p* > 0.05), or treatment × rotations interaction (*F*_(3,48)_ = 0.8517; *p* > 0.05; Fig. 4*C*). Regarding Lesion groups, a two-way ANOVA for repeated measures revealed a significant effect of treatment (*F*_(2.520, 75.59)_ = 6.276; *p* < 0.01), behavior (*F*_(1,30)_ = 29.85; *p* < 0.0001), and treatment × behavior interaction (*F*_(3,90)_ = 7.592; *p* < 0.001; Fig. 4*C*). When treated with intracollicular microinjections of physiological saline, 6-OHDA rats exhibited an increased number of APO-induced contraversive rotations (Bonferroni’s multiple-comparisons test; *p* < 0.05), revealing a clear asymmetry toward the opposite side of the lesion. This asymmetry was still observed after ipsilateral (*p* < 0.05) and contralateral (*p* < 0.01), or even bilateral (*p* < 0.05) MK-801 microinjections in the IC. In addition, the number of contralateral rotations from the 6-OHDA groups was analyzed in more detail. A one-way ANOVA was performed, revealing a significant effect of treatment (*F*_(3,60)_ = 3.933; *p* < 0.05) and, according to Bonferroni’s multiple-comparisons test, the number of contraversive rotations from MK-801 in the contralateral IC-treated group was higher compared with those displayed by the physiological saline-treated group (*p* < 0.05), suggesting a potentiation of asymmetry.

A two-way ANOVA for repeated measures was used to compare the number and direction of rotations induced by APO after NMDA was microinjected into the IC. Regarding Sham lesion groups, there was no significant effect of treatment (*F*_(1.312,30.17)_ = 3.521; *p* > 0.05), behavior (*F*_(1.46)_ = 0.2040; *p* > 0.05), or treatment × behavior interaction (*F*_(2,46)_ = 0.2065; *p* > 0.05), providing no evidence of asymmetry after physiological saline or NMDA microinjections into the IC (ipsilaterally or contralaterally to the lesion; Fig. 4*D*). In addition, the behavior of 6-OHDA rats that received NMDA microinjected into the IC after APO injection, was analyzed. A two-way ANOVA for repeated measures revealed a significant effect of behavior (*F*_(1,18)_ = 23.38; *p* < 0.0001) but no effect of treatment (*F*_(1.606,_ 27.30 = 0.3503; *p* > 0.05) or behavior × treatment interaction (*F*_(2,34)_ = 0.7981; *p* > 0.05). Bonferroni’s multiple-comparisons test revealed that lesioned rats receiving physiological saline into the IC exhibited more contraversive rotations (*p* < 0.05), indicating a clear asymmetry as a consequence of lesion and APO treatment. NMDA microinjected into the IC (both ipsilateral or contralateral) did not affect this asymmetry, since after this treatment 6-OHDA rats still exhibited more contraversive rotations (*p* < 0.05; Fig. 4*D*).

### TH immunoreactivity in the SNpc

The immunohistochemical analysis showed that the 6-OHDA lesions (right MFB) led to a substantial loss of TH-labeled cells (mean loss, 86.91 ± 2.10%) in the SNpc compared with the contralateral No lesion side. A representative image comparing the lesion and the intact side of the same rat is shown in Figures 3*E* and 4*G*. A one-way ANOVA revealed that the 6-OHDA groups showed significantly fewer TH-labeled cells in the SNpc compared with the Sham lesion group (*F*_(2,17)_ = 181.5; *p* < 0.0001; Fig. 3*D*), but no difference in the number of labeled TH cells in the SNpc of 6-OHDA rats and intracollicular electrode implant in the IC. Regarding the 6-OHDA rats with an intracollicular cannula implanted in the IC, an unpaired Student’s *t* test revealed that TH density in the SNpc was significant less for the Lesion group compared with the Sham lesion group (*t* = 35.66, df = 26, *p* < 0.0001; Fig. 4*F*).

### Electrode tip and microinjection cannula placement

Placements of electrode tips or microinjection sites in the IC are shown in Figures 3*D* and 4*E*, respectively. The electrode or cannula tips were situated in the central nucleus of the IC. The number of points is less than the total number of animals studied because of graphical overlapping.

### Intrastriatal neural activity after microinjection of either MK-801 or NMDA into the IC

**Figure 5.**
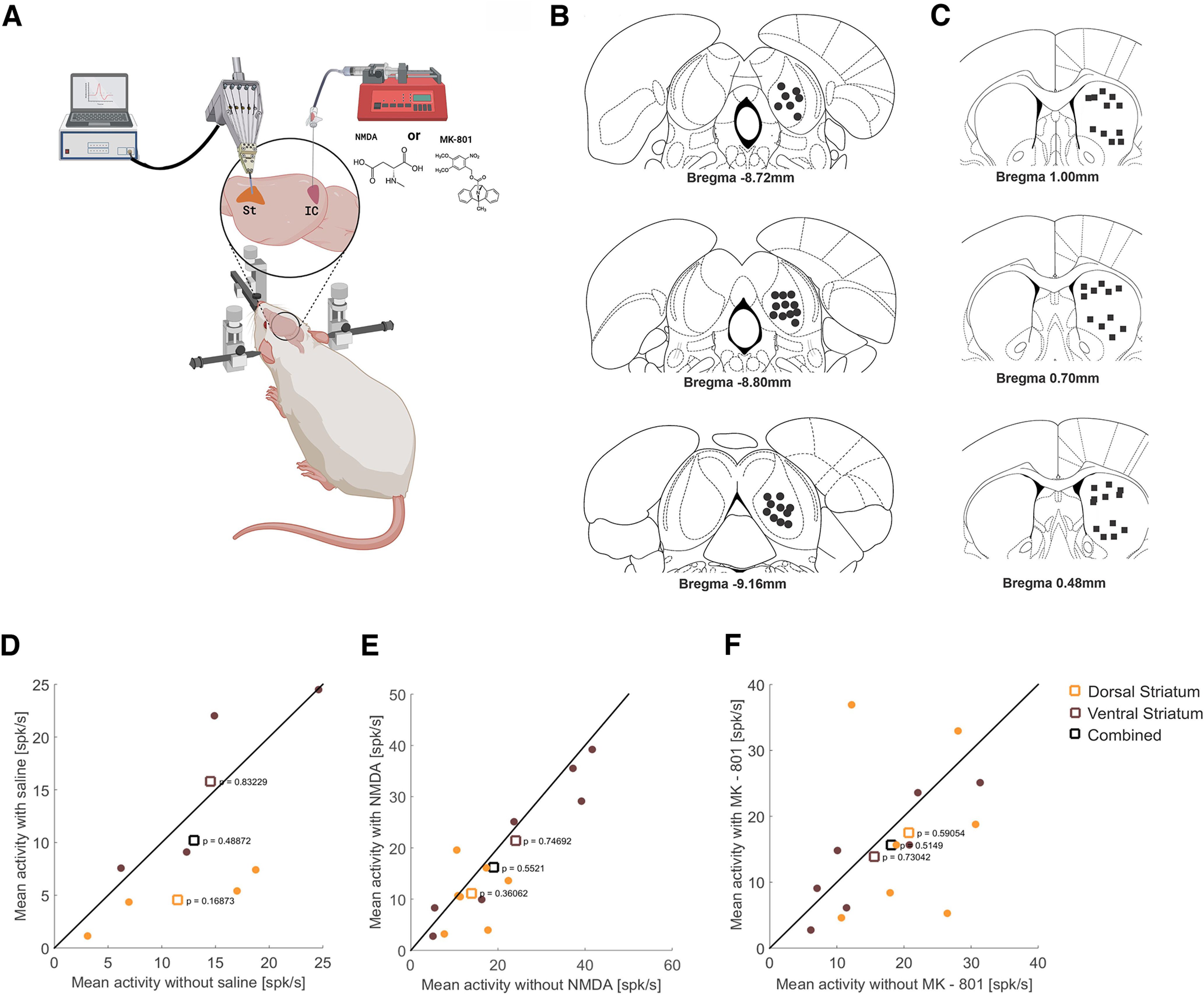
Recording of intrastriatal neural activity after microinjection of either MK-801 or NMDA in the IC of anesthetized rats. ***A***, Graphical representation summarizing the electrophysiological procedure (created with BioRender.com). The Mini Matrix was lowered until the tip of each tube rested just above the dural surface, and the electrodes were moved into the brain aiming at the neostriatum. Neural recordings were made from two different depths (dorsal and ventral striatum; baseline measurements) before and after NMDA, MK-801, or physiological saline was microinjected into the IC. ***B***, ***C***, Location of microinjection sites in the IC (black points) and recording sites in the neostriatum (black squares) in cross-section diagrams of the atlas of Paxinos and Watson (2007). ***D***–***F***, Intracollicular microinjection of physiological saline, NMDA, or MK-801 did not affect the mean firing rate (in spikes per second) at the two different penetration depths in the neostriatum (two-sample *t* tests). Intracollicular microinjection of physiological saline, NMDA, or MK-801 did not affect the mean firing rate (in spikes per second) in the neostriatum as a whole (combined) or at the two different penetration depths (dorsal and ventral striatum; two-sample *t* tests).

To test whether glutamatergic manipulations in the IC affect neural activity in the neostriatum, neuronal activity was recorded from two different depths (considered as more dorsal or more ventral regions of the neostriatum) in anesthetized rats. There was no significant difference in striatal activity (number of spikes) between the two depths for all three microinjection conditions (*t*_(saline3.5)_ = −2.47, *p* = 0.08; *t*_(NMDA8.0)_ = −1.75, *p* = 0.12; *t*_(MK-80110_._31)_ = 0.62, *p* = 0.55; Fig. 5*D–F*). Additionally, there was no significant difference in the neural activity at both depths in the neostriatum assessed after the microinjection of MK-801, NMDA, or physiological saline compared with the respective baseline activity.

During microinjections, an unexpected result was observed. Although the rats were anesthetized and their heads fixed in the stereotaxic apparatus, immediate and sustained protraction of the vibrissae was produced bilaterally by intracollicular microinjection of NMDA but not MK-801 or physiological saline. Retraction was never produced. The effect was temporary, since ∼1 min after microinjection the vibrissae were again retracted.

### Neural tract tracing of pathways connecting the IC to the reticular formation structures

**Figure 6.**
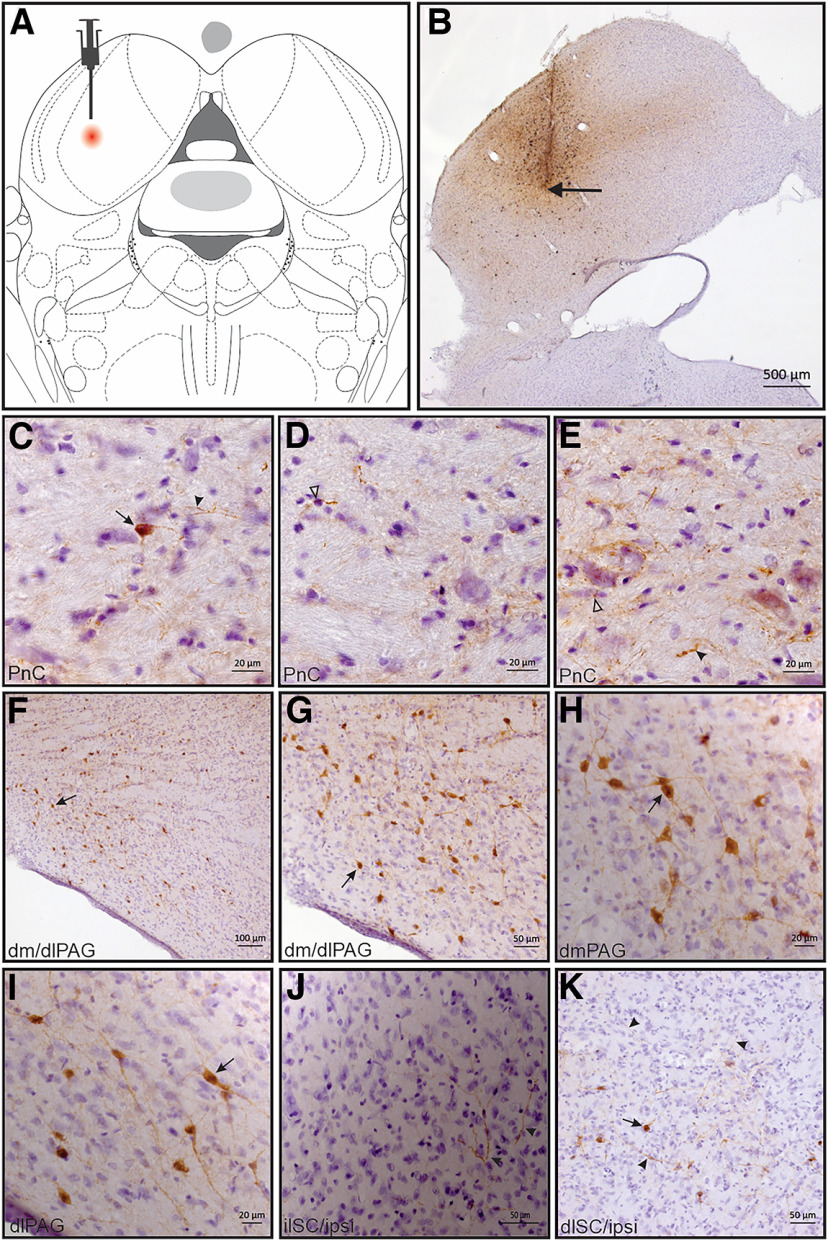
Photomicrographs of transverse sections of Wistar rat brain. ***A***, Diagrammatic representation of a transverse section, showing a histologically confirmed microinjection site (red circle) of the BDA neural tract tracer (3000 MW) in the IC depicted in a modified drawing from the atlas of Paxinos and Watson (2007). ***B***, Wistar rat dorsal midbrain in a transverse section, showing a representative site of BDA microinjection in IC (black arrow). ***C–E***, Transverse sections of the PnC showing BDA-labeled neuronal bodies (black arrow) and axonal fibers (***C***, black arrowheads), terminal buttons (***D***, open arrowheads), and axonal fibers (***E***, black arrowheads) and terminal buttons (open arrowheads) connected to the inferior colliculus. ***F***–***I***, Transverse sections of the dlPAG, ilSC, and dlSC, showing BDA-labeled neuronal bodies (black arrow) in dlPAG reciprocally connected to the IC. ***J***, BDA-labeled axonal fibers (black arrowheads) in ilSC. ***K***, BDA-labeled neuronal bodies (black arrow) and axonal fibers (black arrowheads) situated in the dlSC ipsilateral to BDA deposit sites in the IC.

**Figure 7.**
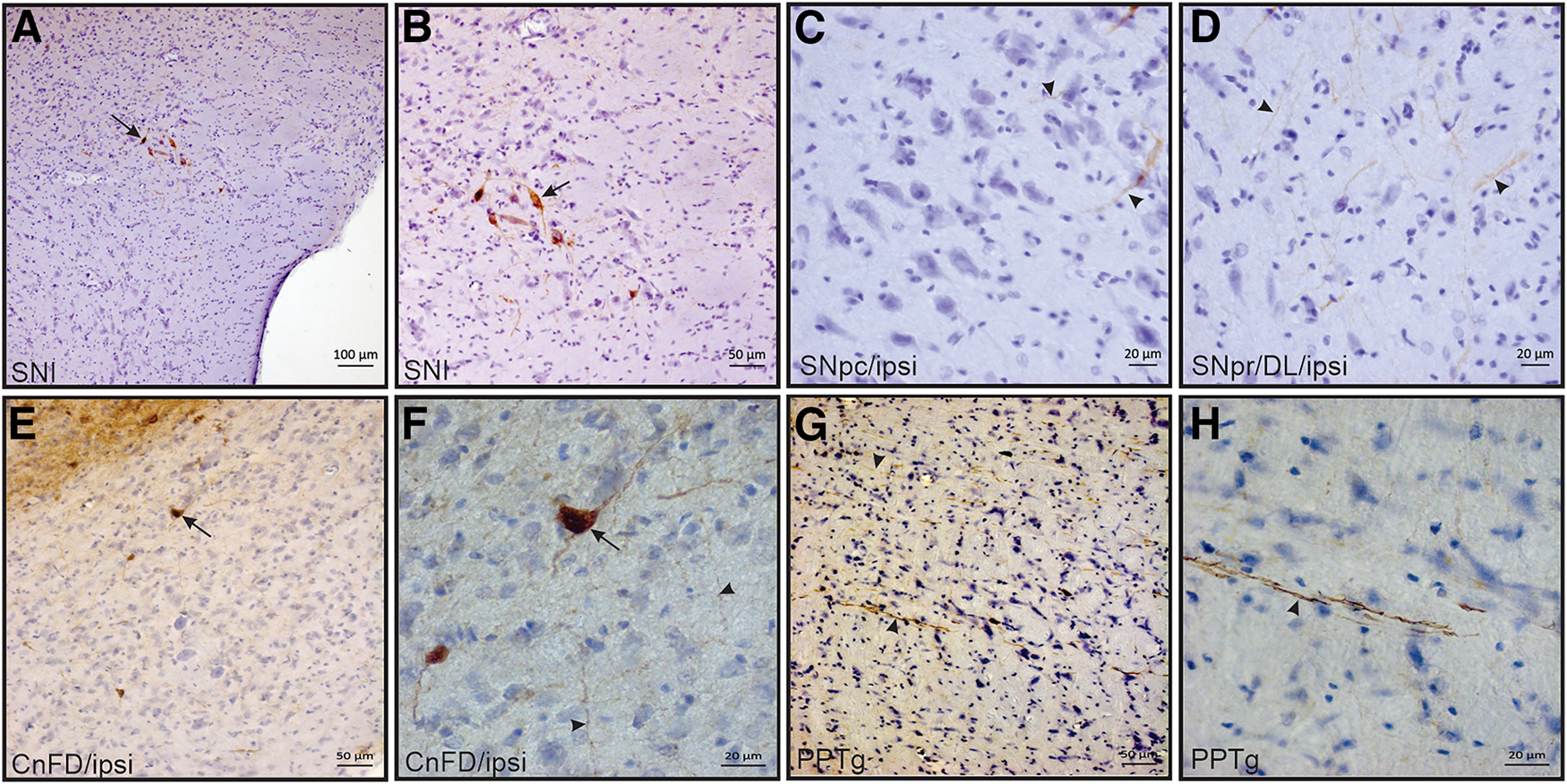
Anatomical investigation. ***A–D***, Transverse sections at the level of SNpl (***A***, ***B***), SNpc (***C***), and SNpr (***D***), showing BDA-labeled neuronal bodies (black arrow) in SNpl, axonal fibers (***C***, black arrowheads) in SNpc, and (***D***) axonal fibers (***D***, black arrowheads) in SNpr ipsilaterally to BDA deposit sites in the IC. ***E***, ***F***, Photomicrographs of transverse sections showing BDA-labeled neuronal bodies (black arrow) and axonal fibers (black arrowheads) in the CnFD ipsilaterally connected to the IC. ***G***, ***H***, BDA-labeled axonal fibers (black arrowheads) in ipsilateral PPTg.

Figure 6, *A* and *B*, shows the microinjection site of the bidirectional neural tract tracer BDA into the IC. Cell bodies, neuronal fibers, and terminal buttons were found in the caudal part of the pontine reticular nucleus (PnC; see Fig. 6*C–E*). Neuronal perikarya were also found in the dorsomedial columns of the periaqueductal gray matter (PAG) and dorsolateral columns of the PAG (dlPAG; Fig. 6*F–I*). Neuronal fibers were found in the ipsilateral intermediate layers of the superior colliculus (ilSC; Fig. 6*J*), and both BDA-labeled perikarya and neuronal fibers were found ipsilaterally in the deep layers of the superior colliculus (dlSC; Fig. 6*K*). It was also found that BDA-labeled neuronal cell bodies in the substantia nigra pars lateralis (Fig. 7*A*,*B*), in addition to neuronal fibers ipsilaterally located in the SNpc (Fig. 7*C*) and in the SN pars reticulata (SNpr) near the SNpl (Fig. 7*D*). Finally, BDA-labeled cell bodies and axonal fibers were ipsilaterally found in the dorsal part of the cuneiform nucleus (CnFD; Fig. 7*E*,*F*), in addition to BDA-labeled axonal fibers spread in the pedunculo-pontine tegmental nucleus (PPTg; Fig. 7*G*,*H*). The neurotracer microinjections in the IC also labeled the well known connections with auditory structures (data not shown), such as the dorsal and ventral cochlear nuclei (cell bodies found in ventral anterior division), intermediate nucleus of the lateral lemniscus nucleus (labeled cell bodies and fibers), medial geniculate nucleus (BDA-labeled fibers and terminal buttons), sub-brachial nucleus (cell bodies and fibers), medial geniculate nucleus, and superior olive complex (labeled cell bodies and terminal buttons in medial superior olive, lateral superior olive, and superior paraolivary nucleus).

## Discussion

Although it has been previously demonstrated the efficacy of intracollicular glutamatergic compounds or DBS in improving haloperidol-induced catalepsy in rats (Melo et al., 2010; Medeiros et al., 2014; Melo-Thomas and Thomas, 2015; Tonelli et al., 2018; Ihme et al., 2020), this animal model suffers from a lack of chronic behavioral motor deficits that represent the cardinal features of PD in humans. To have some insight into the potential role of the IC on improving chronic motor deficits, the present study analyzed whether these treatments influence spontaneous or APO-induced rotations, in rats with unilateral infusion of the dopamine neurotoxin 6-OHDA. In this study, unilateral 6-OHDA lesions aimed at the MFB produced a marked degeneration of the DAergic neurons in the SNpc with >80% of neuronal loss. The EPM test, classically used to assess anxiety, was used to investigate lesion-induced spontaneous asymmetry. For that purpose, the EPM test was conducted under low light intensity to reduce anxiety and encourage exploration. Indeed, no anxiety was observed in both groups (sham lesioned and lesioned), since there was no difference in the times spent and numbers of entries in the open and closed arms. This was particularly important since it has been reported that unilateral 6-OHDA lesions can have anxiogenic effects when the rats are tested under more bright light conditions (O’Connor et al., 2016). Thus, the conditions under which the present EPM test was conducted abolished the possible anxiogenic effect of the 6-OHDA lesion and therefore promoted exploration on the open arms. Indeed, the total number of turns, half-rotations, and rotations were not different when comparing lesioned and sham-lesioned groups. However, these behaviors were biased toward the damaged hemisphere, revealing a clear and expected ipsiversive asymmetry, while sham-lesioned rats showed no preference for any side. This result is coherent with a previous study conducted in intact rats (Schwarting et al., 1991), showing that the number of turns toward one side of the body is highly correlated with the amount of thigmotactic scanning (locomotion along the wall with the vibrissae in contact with it) performed with the other side of the body. Thus, hemiparkinsonian rats scan more with the side contralateral to the lesion; that is, the side that should show sensory neglect. This can also explain the higher incidence of ipsilateral risk assessments performed by lesioned rats at the present study, since Garcia et al. (2005) suggested that, in normal rats, behaviors other than the number of entrances on the arms at the EPM are modulated by thigmotactic cues, rather than visual ones. Altogether, these results strengthen the participation of a component of thigmotaxis modulating asymmetry and suggest, for the first time, that the EPM test may be useful in research on behavioral asymmetries/thigmotaxis.

Although the experimental condition was not anxiogenic, when receiving intracollicular 30 Hz DBS, the 6-OHDA rats spent more time in the open arms. This result is in accordance with our previous data showing an anxiolytic effect of intracollicular 30 Hz DBS in intact rats (Ihme et al., 2020). The number of risk assessments, an ethological measure often sensitive to drug action (Holmes and Rodgers, 1998; Coimbra et al., 2017; Paschoalin-Maurin et al., 2018), was not affected by 30 Hz DBS. In addition, DBS did not affect the number of turns, half-rotations, or rotations in sham control rats, or the ipsiversive asymmetry induced by the unilateral 6-OHDA lesion revealed in the EPM test. On the other hand, when the rats were challenged with APO and submitted to an open field test arena, a different effect of 30 Hz DBS was observed. Both lesion groups exhibited more contraversive rotations compared with sham-lesioned groups, and the incidence of these rotations was not influenced by 30 Hz DBS. However, when taking into account the specific site of electrode placement within the IC, 30 Hz DBS in the vCN, but not in the dCN, attenuated the contraversive asymmetry induced by the DA receptor agonist. More than that, this attenuation was dependent on the side of stimulation, since the number of contralateral rotations was reduced only when the electrode was placed in the IC vCN ipsilateral to 6-OHDA lesion. This is particularly important since it was already demonstrated that intracollicular electrical stimulation in the vCN, but not in the dCN, induced immediate motor responses in anesthetized rats (Melo-Thomas and Thomas, 2015). Thus, the results of the present study support the hypothesis that intracollicular 30 Hz DBS affects contraversive body asymmetry induced by unilateral 6-OHDA lesion in rats challenged with APO and suggest that this effect depends on the placement of the electrode tip in the IC. The reason why intracollicular 30 Hz DBS improves APO-induced asymmetry but not spontaneous asymmetry is not clear. The main difference between both conditions is the activation or nonactivation, respectively, of upregulated DA receptors in the DA-deprived hemisphere. The mechanism by which ipsilateral 30 Hz DBS in the IC compensates upregulation of DA receptors there needs to be investigated.

In addition, the present study showed that spontaneous ipsiversive behavior asymmetry (pre-APO condition) induced by a unilateral 6-OHDA lesion is influenced by the glutamatergic neural network in the IC. It was demonstrated that the ipsiversive asymmetry disappeared after microinjection of the glutamatergic antagonist MK-801 into the IC (i.e., there was no difference when comparing the number of ipsiversive and contraversive rotations performed by 6-OHDA rats, regardless of the side of IC microinjection (ipsilaterally, contralaterally, or bilaterally to the lesion). The absence of asymmetry was also observed when the glutamatergic agonist NMDA was microinjected in the IC contralateral to lesion. Surprisingly, NMDA microinjected in the IC ipsilateral to lesion produced a clear contraversive asymmetry (i.e., it reverted the lateralization). Importantly, there was no difference when comparing the number of ipsiversive or contraversive spontaneous rotations after IC microinjection of MK-801, NMDA, or saline into rats with sham lesions (i.e., no effect of IC treatment or any asymmetry was observed).

When challenged with APO, 6-OHDA rats receiving physiological saline in the IC exhibited an increased number of contraversive rotations, revealing a clear asymmetry toward the side opposite to that of the lesion. Here, it was demonstrated that the APO-induced contraversive asymmetry in 6-OHDA rats can be potentiated by MK-801 microinjected into the IC contralateral to lesion side, whereas asymmetry was unaffected when the microinjection was performed bilaterally or in the ipsilateral IC. In contrast, NMDA microinjected into the IC, ipsilateral or contralateral to lesion side, did not affect contraversive asymmetry induced by APO in lesioned rats.

Together, the present results support our hypothesis that the hypersensitivity caused by neurochemical lesion of nigrostriatal DAergic pathway can be influenced by IC 30 Hz DBS or glutamatergic intracollicular neurotransmission. Further, our findings suggest that the side of the IC receiving 30 Hz DBS or NMDA microinjections with respect to the lesion is particularly relevant. Although it is well known that rotatory behavior induced by 6-OHDA-produced unilateral lesion reflects a striatal DAergic misbalance (Dewar et al., 1997; DeLong and Wichmann, 2007) between the two hemispheres to initiate movements (intact side during the pre-APO condition, lesion side during the post-APO condition), the mechanism by which the glutamatergic neural networks in the IC affects this misbalance is not known; that is, how MK-801 microinjected into the contralateral IC potentiates contralateral rotations in the post-APO condition, but compensated the misbalance between the right and left side in the pre-APO condition regardless of the IC side. It is also not clear how NMDA microinjected into the ipsilateral IC inverted the asymmetry, from ipsiversive to contraversive rotations during the pre-APO condition. Searching to explain these results, it seemed plausible to look at the circuitry connecting the IC with structures involved with motor-related structures. Among these structures, the neostriatum was chosen based on results from the present and previous studies from our group demonstrating the participation of the IC in improving motor impairments induced by (1) MFB 6-OHDA lesion, where unilateral motor deficit correlates well with the degree of the nigrostriatal pathways neurotoxic lesion (Ungerstedt and Arbuthnott, 1970; Schallert et al., 2000; Dowd et al., 2005); and (2) haloperidol-induced catalepsy, where motor deficits occur because of a temporary blockage of striatal DA receptors (Hornykiewicz, 1973; Trevitt et al., 2009; Melo et al., 2010; Medeiros et al., 2014; Melo-Thomas and Thomas, 2015; Tonelli et al., 2018; Ihme et al., 2020). However, contrary to our hypothesis, the results from the present electrophysiological investigation showed that the firing rate and number of spikes in two different depths of the neostriatum remained unchanged after microinjection of NMDA or MK-801 into the IC in anesthetized rats. Since neither the blockade nor the activation of glutamatergic receptors in the IC influenced striatal neural activity in anesthetized rats, but affected contraversive and ipsiversive rotations of rats with unilateral 6-OHDA lesions, as demonstrated in this study, in addition to inducing paradoxical kinesia in cataleptic rats (Melo et al., 2010; Medeiros et al., 2014; Tonelli et al., 2018), one can assume that IC DBS or microinjections of parkinsonian-like rats were effective in improving motor deficits by recruiting motor programs other than the neostriatum.

Indeed, the neural tract tracings performed in this study demonstrated connections between the IC and both dorsal (deep layers of the superior colliculus and dorsal columns of the periaqueductal gray matter) and ventral (SNpl) midbrain structures. Both the deep layers of the superior colliculus and the dorsolateral columns of the periaqueductal gray matter are interconnected (Ribeiro et al., 2005; Freitas et al., 2005), receive inputs from the substantia nigra (Eichenberger et al., 2002; Ribeiro et al., 2005; Castellan-Baldan et al., 2006), a ventral midbrain structure also connected to the neostriatum (Castellan-Baldan et al., 2006; Matias et al., 2019), and send projections to the deep midbrain and pontine reticular formation (Coimbra et al., 2006). These former areas also include the mesencephalic locomotor region (MLR; Sébille et al., 2017), an important midbrain structure implicated in commands for movement initiation and gait selection (Jordan et al., 2008; Takakusaki, et al., 2016). The MLR is composed of structures such as the CnF and pedunculo-pontine nucleus (PPN), which are also reached by axons from the IC neurons, as shown in our neuroanatomical experiment, and both brainstem structures are involved in gait control. Corroborating our anatomic results, optogenetic studies showed that glutamatergic CnF neurons receive massive projections from the IC (Caggiano, et al., 2018). Here it is important to emphasize that the electrical stimulation of both the CnF and the IC can evoke persistent locomotion (Shik et al., 1969) and escape responses (Lovick, 1993; Brandão et al., 1999), respectively. Interestingly, the caudal mesencephalic reticular formation, specifically the PPN, controls locomotion and the awake state, and Goetz et al. (2019) demonstrated in primates that DA depletion did not alter the mean firing rate of noncholinergic caudal mesencephalic reticular formation neurons, but only decreased activity of putative cholinergic PPN neurons. Therefore, it can be speculated that, to compensate behavioral asymmetry in 6-OHDA rats in our study, the IC may be recruiting some alternative downstream motor pathways such as, for instance, the MLR. This hypothesis will be investigated in further studies by our team.

Noteworthy, in this study, both ipsilateral and bilateral vibrissae protractions were observed during NMDA, but not MK-801 or physiological saline, microinjection in the IC of anesthetized rats. Vibrissae movements (as well as tail, trunk, and leg movements) during electrical stimulation of the IC in anesthetized rats have been reported by our group (Melo-Thomas and Thomas, 2015). The anatomic bases of such whisking behavior have been described previously (Kis et al., 2004; Nguyen and Kleinfeld, 2005; Kleinfeld et al., 2006). Whisking behavior is commonly used by rats to actively explore their environment (Gibson, 1962; Gamzu and Ahissar, 2001), especially during potentially dangerous situations (Arkley et al., 2014; Filgueiras et al., 2014). Since the IC has been related to anxiety-like behavior (Brandão et al., 1993, 1994; Melo and Brandão, 1995), it seems plausible to think that some kind of multisensory (somatosensory–auditory–motor) integration should occur at the mesencephalic level during emergency situations to generate a most appropriate motor output (Huffman and Henson, 1990; Young et al., 1995; Kanold and Young, 2001; Shore, 2005). Our findings represent a step forward aiming to understand this kind of multisensory integration influencing whisking behavior by suggesting that glutamatergic neurotransmission in the IC may be involved.

Data presented here and previous findings strengthen the assumption that the IC plays an important role in mammalian motor control. Therefore, the IC may provide a critical site to improve motor impairments because of basal nuclei damage, recruiting other structures involved in motor function bypassing the neostriatum. Understanding how glutamatergic mechanisms in the IC influence motor control, classically attributed to the basal nuclei circuitry, could be useful in the development of new therapeutics to treat PD and other motor disorders.

## Synthesis

Reviewing Editor: Mikko Airavaara, Helsingin yliopisto Decisions are customarily a result of the Reviewing Editor and the peer reviewers coming together and discussing their recommendations until a consensus is reached. When revisions are invited, a fact-based synthesis statement explaining their decision and outlining what is needed to prepare a revision will be listed below. The following reviewer(s) agreed to reveal their identity: Nicole Chambers, Stefan Schuster.

The ideas within the manuscript are innovative in that the author is investigating the contribution of the inferior colliculus (IC) to lateralization of behavior following 6-OHDA lesion as assessed through elevated plus maze and apomorphine rotations, and no one really investigates the IC in Parkinsonian models. To do so the authors use a moderately lesioned hemiparkinsonian 6-OHDA rat model that received injections into the right medial forebrain bundle and authors used unilateral IC deep brain stimulation (DBS) or microinfusion of the glutamate NMDA antagonist MK-801 or the agonist NMDA into the IC. Authors also assess striatal activity during anesthesia after microinjection of MK-801 or NMDA into IC. Finally, authors show results of microinjection of a bidirectional neural tracer BDA into the IC. Although the ideas underlying the manuscript are interesting and innovative, I have multiple concerns regarding this manuscript which appear below:

Major:

1) Testing for animals starts 1 week after 6-OHDA lesion surgery, which means that the lesion has not yet stabilized (Jeon et al., 1995). Furthermore, research exists in which apomorphine is shown to be protective in the MPTP and 6-OHDA models.

2) Nowhere in the manuscript is it stated that animals assigned to the NO DBS and DBS conditions were compared for lesion efficacy. At a bare minimum, there should be some behavioral assessment that was conducted to judge motor deficits and then animals should have been assigned to equally lesioned groups using this pre-assessment. Otherwise, it becomes difficult to judge whether results that were obtained were because of unequal DA lesions in rats. This could potentially be remedied post-hoc by adding a figure comparing lesion efficacy (percent DA cell loss in SNc) across the conditions for lesioned animals.

3) According to the manuscript, lesioned DBS animals are further subdivided into 4 more groups based on whether cannula is implanted ipsilateral to lesion or contralateral to lesion as well as whether the tip of the electrode was located in the dCN or vCN. Was this also done for sham animals? It should be explicitly stated how many animals were in each of these groups. At present with the information that is given, it seems that there would not be enough animals to fully power this experiment.

4) Were any animals excluded due to the cannula not being in the right structure? Were animals excluded from analysis if they did not reach a certain percentage of lesion?

5) Some animals from the first experiment were reused for Apomorphine rotations-how were these chosen? What groups were they from?

Minor

6) It seems that for experiment 1 that animals with lesion receiving DBS are moving slightly less than lesioned animals not receiving DBS based on the number of entries being reduced. Is it possible that stimulation in the IC is inducing auditory percepts that are causing animals to move less overall? If this were true IC DBS as a therapy for PD would be untenable as it would exacerbate PD akinesia

7) If possible, authors should cite references that use the same or similar doses of NMDA and MK801 NMDA: 30 nmol / 0.5 uL MK-801 : 30 nmol/0.5 uL

8) If possible, authors should cite references for their dose of apomorphine, which seems quite high in comparison to most literature which uses 0.05 mg/kg - 0.2 mg/kg.

9) What was the spread of the microinjections into IC? Did any of the microinjections cross the boundaries into other structures either in the NMDA/MK-801 study or in the BDA tracing study?

10) Is it possible that DBS stimulation partially worked by crossing the boundaries of the IC and affecting other brain areas?

11) Was DBS stimulation turned on before testing? In many brain structures it takes at least 1 hr of on time to see full effects of DBS

12) Missing definition of risk assessment in EPM-this is not defined in methods and suddenly appears in results.

13) Discrepancy in description of electrode implant in rats. At beginning of results it is stated that rats were implanted with a bilateral electrode; whereas, in the methods it states that rats were implanted unilaterally with a microelectrode.

14) Why are the graphs in Figure 4 labeled “Narrow Rotations”? 15) Labeling of graphs with graph titles could help with clarity and consistency in the manuscript.

16) In Figure 5 what are the combined dots in Figures D, E, and F? Also the figures are referenced incorrectly in the figure caption for the microinjections-they are referred to as E-G when they are actually D-F.

17) How were the numbers of subjects determined a priori, especially for the neural tracer study which only contained 4 animals. Also are these projections still intact in the PD model?

## Author Response

Dear Prof. Dr. Mikko Airavaara

The authors appreciate and are grateful to the referee’s evaluation and encouragement concerning our study. We thank the comments that were useful for the improvement of the manuscript.

Please find bellow the answers addressing all point-by-point reviewers’ comments.

Answers to the reviewers’ comments

Response letter - e-Neuro

Synthesis Statement for Author (Required):

The ideas within the manuscript are innovative in that the author is investigating the contribution of the inferior colliculus (IC) to lateralization of behavior following 6-OHDA lesion as assessed through elevated plus maze and apomorphine rotations, and no one really investigates the IC in Parkinsonian models. To do so the authors use a moderately lesioned hemiparkinsonian 6-OHDA rat model that received injections into the right medial forebrain bundle and authors used unilateral IC deep brain stimulation (DBS) or microinfusion of the glutamate NMDA antagonist MK-801 or the agonist NMDA into the IC. Authors also assess striatal activity during anesthesia after microinjection of MK-801 or NMDA into IC. Finally, authors show results of microinjection of a bidirectional neural tracer BDA into the IC. Although the ideas underlying the manuscript are interesting and innovative, I have multiple concerns regarding this manuscript which appear below:

Major:

1) Testing for animals starts 1 week after 6-OHDA lesion surgery, which means that the lesion has not yet stabilized (Jeon et al., 1995). Furthermore, research exists in which apomorphine is shown to be protective in the MPTP and 6-OHDA models.

Response: The authors thank the reviewers for raising this issue and mention the interesting paper from Jeon et al (1995). However, reading that paper we noticed that fiber degeneration was not observed after post lesion day 7 and the level of neuron death appears to be maximal between days 4 and 6 post-surgery (see Fig 1, Jeon et al., 1995) indicating that the lesion is already stablished. The methodology used in our present study was based on previous studies (Gregorio et al., 2009; Da Cunha et al., 2008; Adu and Mabandla, 2019) in which rats were challenged with apomorphine 1 week after 6-OHDA was infused into medial forebrain bundle.

Probably the referees mean therapeutic effect, instead protective effect as demonstrated by Carbone et al. (2019), and Deley et al. (2004). It is true that apomorphine (as a D1- and D2-like receptor agonist) has a therapeutic effect and can relieve PD patients from motor complications observed during “off” periods in advanced phase of the disease. However, for that, apomorphine should be infused continuously which was not the case in our study. Apomorphine challenges, as used by us have long been used as a tool to induce rotations and test for lateralization at rats receiving unilateral injection of 6-OHDA in substantia nigra or medial forebrain bundle, a classical animal model for Parkinsonism.

References:

2 Gregório M L, Wietzikoski E C, Ferro M M, Silveira J L M, Vital M A B F, Da Cunha C. Nicotine induces sensitization of turning behavior in 6-hydroxydopamine lesioned rats. Neurotox Res. 2009 May;15(4):359-66. doi: 10.1007/s12640-009-9041-1.

Da Cunha C, Wietzikoski EC, Ferro MM, Martinez GR, Vital MA, Hipólide D, Tufik S, Canteras NS. Hemiparkinsonian rats rotate toward the side with the weaker dopaminergic neurotransmission. Behav Brain Res. 2008 Jun 3;189(2):364-72. doi: 10.1016/j.bbr.2008.01.012.

Adu TS, Mabandla MV. Effects of bromelain on motor responses following intra-medial forebrain bundle 6-OHDA injection in rat model of parkinsonism. Metab Brain Dis. 2019 Dec;34(6):1557-1564. doi: 10.1007/s11011-019-00462-9.

Carbone F , Djamshidian A , Seppi K , Poewe W. Apomorphine for Parkinson’s Disease: Efficacy and Safety of Current and New Formulations. CNS drugs. 2019 Sep;33(9):905-918. doi: 10.1007/s40263-019-00661-z.

Deleu D, Hanssens Y, Northway MG.Subcutaneous apomorphine : an evidence-based review of its use in Parkinson’s disease.

Drugs Aging. 2004;21(11):687-709. doi: 10.2165/00002512-200421110-00001.PMID: 15323576 Review.

2) Nowhere in the manuscript is it stated that animals assigned to the NO DBS and DBS conditions were compared for lesion efficacy. At a bare minimum, there should be some behavioral assessment that was conducted to judge motor deficits and then animals should have been assigned to equally lesioned groups using this pre-assessment. Otherwise, it becomes difficult to judge whether results that were obtained were because of unequal DA lesions in rats. This could potentially be remedied post-hoc by adding a figure comparing lesion efficacy (percent DA cell loss in SNc) across the conditions for lesioned animals.

Response: Based on histological staining for tyrosine hydroxylase, only rats presenting a marked degeneration of the DAergic neurons in the SNpc with more than 80% of neuronal loss were included in our study. For information about the lesion please see Fig. 3D and a statement in the Discussion, lines 7-8. However, in order to highlight this information, and clarify about animals’ assignment, a second statement was added on Pg 9, lines 14-16, as follow:

The rats were aleatorily assigned into NoDBS and DBS groups. Only rats presenting a marked degeneration of the DAergic neurons in the SNpc with more than 80% of neuronal loss were included in the present study.

3) According to the manuscript, lesioned DBS animals are further subdivided into 4 more groups based on whether cannula is implanted ipsilateral to lesion or contralateral to lesion as well as whether the tip of the electrode was located in the dCN or vCN. Was this also done for sham animals? It should be explicitly stated how many animals were in each of these groups. At present with the information that is given, it seems that there would not be enough animals to fully power this experiment.

Response: Data from sham lesion rats were not compared considering electrode placement in the IC as a factor, because the total amount of rotations for each rat was close to zero. To better clarify the number of animals by group the following statement was added on Pg.8, lines 15-18: "During data analysis, lesion rats were assigned as follows: dCN n=6 and vCN n=12; then, vCN rats were reassigned to contralateral and ipsilateral to lesion (n=6 each). Sham lesion rats were not compared considering electrode placement in the IC as a factor, because the total number of rotations for each rat was close to zero.”

4) Were any animals excluded due to the cannula not being in the right structure? Were animals excluded from analysis if they did not reach a certain percentage of lesion?

Response: Indeed, a total of six rats were excluded from our study and this information was incorporated in the text as follows:

The sentence stating the total number of rats used in the study was replaced by “... One hundred-twenty-six male

Wistar rats...” (see pg 5, line 29).

The following sentence was added on Pg 4, last line and Pg 5 first line:

"A total of six rats was excluded from our study due to electrode misplacement or because they did not present contraversive rotations after apomorphine.”

5) Some animals from the first experiment were reused for Apomorphine rotations-how were these chosen? What groups were they from?

Response: They were chosen aleatorily from sham lesion and lesion groups.

3 Minor

6) It seems that for experiment 1 that animals with lesion receiving DBS are moving slightly less than lesioned animals not receiving DBS based on the number of entries being reduced. Is it possible that stimulation in the IC is inducing auditory percepts that are causing animals to move less overall? If this were true IC DBS as a therapy for PD would be untenable as it would exacerbate PD akinesia.

Response:

The total number of entries into both open and closed arms performed by rats receiving No DBS or DBS did not differ statistically.

It is hard to say whether the IC stimulation is inducing some kind of auditory percepts. In a previous study (conducted under classical aversive conditions), we showed that rats receiving intracollicular 30HZ DBS spent more time and entered more into the open arms indicating an anxiolytic effect (Ihme et al., 2020). This effect would be desirable for PD patients in which anxiety is a common co-morbidity.

7) If possible, authors should cite references that use the same or similar doses of NMDA and MK801 NMDA: 30 nmol / 0.5 uL MK-801 : 30 nmol/0.5 uL

Response: These doses were based on previous studies from our group. The following sentence was added (Pg 5, lines 19-20) indicating references that used the same doses of NMDA, MK-801 and also of apomorphine:

The drugs and doses used here were based on previous studies (Melo et al., 2010; Medeiros et al., 2014; Tonelli et al., 2018; Ciucci et al., 2007).

8) If possible, authors should cite references for their dose of apomorphine, which seems quite high in comparison to most literature which uses 0.05 mg/kg - 0.2 mg/kg.

Response: The authors thank the referees for this remark. Actually, a large range of apomorphine doses up to 1.0 mg/kg (please see da Cunha et al., 2008) to induces rotatory behavior after 6-OHDA lesion, can be found in the literature. The dose used at the present study (0.5 mg/Kg) was based on Ciucci et al., 2007. The reference for apomorphine dose was added on Pg 5, lines 19-20, and the reference Ciucci et al., 2007 was added to the reference list, Pg 25, lines 3-5.

References:

Da Cunha C, Wietzikoskia EC, Ferro MM, Martinez GR, Vital MABF, Hipólide D, Tufik S, Canteras NS (2008) Hemiparkinsonian rats rotate toward the side with the weaker dopaminergic neurotransmission. Behav. Brain Res. 189(2, 3): 364-372.

Ciucci MR, Ma ST, Fox C, Kane JR, Ramig LO, Schallert T. Qualitative changes in ultrasonic vocalization in rats after unilateral dopamine depletion or haloperidol: a preliminary study. Behav Brain Res. 2007 Sep 4;182(2):284-9. doi: 10.1016/j.bbr.2007.02.020.

9) What was the spread of the microinjections into IC? Did any of the microinjections cross the boundaries into other structures either in the NMDA/MK-801 study or in the BDA tracing study?

Response: Previous work regarding substances microinjected in the central nervous system suggested that the diffusion into the surrounding tissue has a direct relationship to the volume given (Myers, 1966; Routtenberg, 1972). Based on such evidence, volumes no larger than 0.5 μL (average spread of 1.04 mm, according to Myers, 1966) were used in the present work.

References:

Myers, R.D., 1966. Injection of solutions into cerebral tissue: relation between volume and diffusion. Physiol. Behav. 1, 171-174.

Routtenberg, A., 1972. Intracranial chemical injection and behavior: a critical review. Behav. Biol. 7, 601-641.

10) Is it possible that DBS stimulation partially worked by crossing the boundaries of the IC and affecting other brain areas?

Response: Based on previous pilot studies (not published) performed in anaesthetized rats, we observed that the stimulation spread area is around 600 μm during intracollicular 130 Hz DBS. Since the stimulation frequency in the present study was even slower (30Hz), we expected possible spreading to be smaller (somewhere between 300 and 600 μm), which speaks against crossing the boundaries of the IC.

11) Was DBS stimulation turned on before testing? In many brain structures it takes at least 1 hr of on time to see full effects of DBS

Response: For EPM test, the rats were first stimulated for 5 min in a home cage placed near the EPM.

Thereafter, the rats were placed on the EPM, with stimulation being continued throughout 5 min testing (see description at Pg.7, lines 22-24). So, the total time stimulation for each rat was 10 min and no additional stimulation was performed before testing.

4 For assessing rotatory behavior under intracollicular 30 Hz DBS, immediately after each rat had received APO, the stimulation cable was connected to the implanted electrode and rats were placed into the arena under continuous stimulation during 30 min (see description at Pg 8, lines 10-13). Again, no additional stimulation was performed before testing.

12) Missing definition of risk assessment in EPM-this is not defined in methods and suddenly appears in results.

Response: Probably that was a misunderstanding. Risk assessment was already defined in methods at Pg 8, lines 1-2, as follow: "(iv) number of risk assessment (aborted attempts at entry into open arms, which includes scanning, stretch attend, flat back approach; Griebel et al., 1997)”.

However, we replaced this definition by a more detailed one (Pg.8, lines 1-6): "(iv) number of risk assessments contraversive and ipsiversive to the lesion side. Here, risk assessment was defined as aborted attempts to enter into open arms, which includes scanning, stretch/attend postures (when the rat stretches to its full length with its forepaws, keeps its hind paws in the same place, and then turns back) and flat back approach (locomotion during which the animal stretches to its full length and cautiously moves forward; Griebel et al., 1997; Coimbra et al., 2017; Ferreira-Sgobbi et al., 2022).

Added citations were incorporated in the reference list.

References:

Coimbra NC, Calvo F, Almada RC, Freitas RL, Paschoalin-Maurin T, Dos Anjos-Garcia T, Elias-Filho DH, Ubiali WA, Lobã Soares B, Tracey I. Opioid neurotransmission modulates defensive behavior and fear-induced antinociception in dangerous environments. Neuroscience. 2017; 354:178-195. doi: 10.1016/j.neuroscience.2017.04.032.

Ferreira-Sgobbi R, de Figueiredo RM, Frias AT, Matthiesen M, Batistela MF, Falconi-Sobrinho LL, Vilela-Costa HH, Sá SI, Lovick TA, Zangrossi H Jr, Coimbra NC. Panic-like responses of female Wistar rats confronted by Bothrops alternatus pit vipers, or exposure to acute hypoxia: Effect of oestrous cycle. Eur J Neurosci. 2022;55(1):32-48. doi: 10.1111/ejn.15548.

13) Discrepancy in description of electrode implant in rats. At beginning of results it is stated that rats were implanted with a bilateral electrode; whereas, in the methods it states that rats were implanted unilaterally with a microelectrode.

Response: We thank the referees for this remark. In fact, our statement at the beginning of the results was wrong. Actually, each rat received only one electrode implanted into the right or left IC. The statement was therefore replaced (Pg 12, lines 24-25) as follows:

"Briefly, one week after receiving a unilateral microinjection of 6-OHDA in the MFB and an electrode implanted in the right or left IC...”

14) Why are the graphs in Figure 4 labeled “Narrow Rotations”? 15) Labeling of graphs with graph titles could help with clarity and consistency in the manuscript.

Response: Although narrow rotations were defined in the methods (see Pg 9, line 1), we agree with the referees that this information should not appear on the graph title. Accordingly, Fig. 4 has been changed.

16) In Figure 5 what are the combined dots in Figures D, E, and F? Also the figures are referenced incorrectly in the figure caption for the microinjections-they are referred to as E-G when they are actually D-F.

Response: We thank the referee for these remarks. The legend of Fig. 5 was changed as follow:

The sentence “Intracollicular microinjection of either physiological saline, NMDA or MK-801 did not affect the mean firing rate (spikes/s) at the two different penetration depths in the neostriatum (two-sample t-tests).” Was replaced by: "Intracollicular microinjection of either physiological saline, NMDA or MK-801 did not affect the mean firing rate (spikes/s) in the neostriatum as a whole (combined) or at the two different penetration depths (dorsal and ventral striatum; two-sample t-tests).” "E-G” was replaced by “D-F”.

17) How were the numbers of subjects determined a priori, especially for the neural tracer study which only contained 4 animals. Also are these projections still intact in the PD model?

Response: The neural tract tracer was microinjected in six animals, as performed in other neuroanatomical approaches (Coimbra et al., 2006; Almada et al., 2001). However, some neurotracer deposits (n = 2) were found 5 to be very close to the dorsomedial (reaching the medial part of the dorsal cortex of the inferior colliculus; Bregma = -8.80m) and lateral columns (reaching the medial part of the central nucleus of the inferior colliculus; Bregma = -8.80m) of the periaqueductal gray matter and they were discarded from the neuroanatomical experiments. The morphology of the connections investigated in our study was characterized in intact Wistar rats; therefore, we cannot make a definite statement regarding subjects with 6-OHDA lesions. We assume, however, that the morphology of these connections will not be affected by 6-OHDA microinjection in the nigrostriatal pathway at the level of the medial forebrain bundle, a pathway particularly far from those presently traced. In addition, since dopamine is not the main neurotransmitter involved in auditory pathways (Adams and Mugnaini, 1984; Comis and Leng, 1979; Faingold et al., 1989; Galetti et al., 1987; Koomer and moore, 1980; Thompsom et al., 1995) most likely it may not have been affected by 6-OHDA injection in the present study. This makes our study more interesting, since we are probably, manipulating a preserved pathway to relieve motor impairments in Parkinsonian rats.

References:

Adams JC, Mugnaini E. Dorsal nucleus of the lateral lemniscus: a nucleus of GABAergic projection neurons. Brain Res Bull. 1984;13(4):585-90. doi: 10.1016/0361-9230(84)90041-8.

Almada RC, dos Anjos-Garcia T, da Silva JA, Pigatto GR, Wotjak CT, Coimbra NC. The modulation of striatonigral and nigrotectal pathways by CB1 signalling in the substantia nigra pars reticulata regulates panic elicited in mice by urutu-cruzeiro lancehead pit vipers. Behav Brain Res. 2021;401:112996. doi: 10.1016/j.bbr.2020.112996.

Coimbra NC, de Oliveira R, Freitas RL, Ribeiro SJ, Borelli KG, Pacagnella RC, Moreira JE, da Silva LA, Melo LL, Lunardi LO, Brandão ML. Neuroanatomical approaches of the tectum-reticular pathways and immunohistochemical evidence for serotonin positive perikarya on neuronal substrates of the superior colliculus and periaqueductal gray matter involved in the elaboration of the defensive behavior and fear-induced analgesia. Exp Neurol. 2006;197(1):93-112. doi: 10.1016/j.expneurol.2005.08.022.

Comis SD, Leng G. Action of putative neurotransmitters in the guinea pig cochlea. Exp Brain Res. 1979;36(1):119-28. doi: 10.1007/BF00238472.

Faingold CL, Gehlbach G, Caspary DM. On the role of GABA as an inhibitory neurotransmitter in inferior colliculus neurons: iontophoretic studies. Brain Res. 1989;500(1-2):302-12. doi: 10.1016/0006-8993(89)90326-0.

Galetti G, Calzà L, Galetti R, Giardino L, Zanni M, Balli R. Opiates in the acoustic pathway: immunocytochemical and autoradiographic study. Acta Otorhinolaryngol Ital. 1987;7(4):377-85.

Kromer LF, Moore RY. Norepinephrine innervation of the cochlear nuclei by locus coeruleus neurons in the rat. Anat Embryol (Berl). 1980;158(2):227-44. doi: 10.1007/BF00315908.

Thompson AM, Moore KR, Thompson GC. Distribution and origin of serotoninergic afferents to guinea pig cochlear nucleus. J Comp Neurol. 1995;351(1):104-16. doi: 10.1002/cne.903510110.
